# Upregulation of Succinate Dehydrogenase (SDHA) Contributes to Enhanced Bioenergetics of Ovarian Cancer Cells and Higher Sensitivity to Anti-Metabolic Agent Shikonin

**DOI:** 10.3390/cancers14205097

**Published:** 2022-10-18

**Authors:** Lin Wang, Magdalena Cybula, Maria Rostworowska, Luyao Wang, Patryk Mucha, Magdalena Bulicz, Magdalena Bieniasz

**Affiliations:** Aging and Metabolism Program, Oklahoma Medical Research Foundation, Oklahoma City, OK 73104, USA

**Keywords:** ovarian cancer, succinate dehydrogenase, SDHA, shikonin, metabolism, patient-derived xenograft

## Abstract

**Simple Summary:**

In recent years, targeting tumor specific metabolism has gained an interest as a promising therapeutic strategy. We discovered that the overexpression of mitochondrial enzyme succinate dehydrogenase (SDHA) is highly prevalent in ovarian carcinoma patients and contributes to elevated mitochondrial metabolism in ovarian tumor models. The SDHA overexpressing tumor cells are highly metabolically active, relying on both glycolysis and oxidative phosphorylation in mitochondria to meet their energy requirements. Further, we found that those cells are particularly vulnerable to deprivation of essential nutrients such as glucose and glutamine, which led to a substantial reduction of ATP yield. Lastly, we identified an anti-metabolic compound shikonin, which demonstrated a potent anti-tumor efficacy against SDHA overexpressing ovarian cancer cells. Overall, we strive to advance scientific knowledge by uncovering the previously unappreciated role of SDHA in reprogramming of ovarian cancer metabolism, which holds untapped opportunities for therapeutic intervention.

**Abstract:**

We discovered that the overexpression of mitochondrial enzyme succinate dehydrogenase (SDHA) is particularly prevalent in ovarian carcinoma and promotes highly metabolically active phenotype. Succinate dehydrogenase deficiency has been previously studied in some rare disorders. However, the role of SDHA upregulation and its impact on ovarian cancer metabolism has never been investigated, emphasizing the need for further research. We investigated the functional consequences of SDHA overexpression in ovarian cancer. Using proteomics approaches and biological assays, we interrogated protein content of metabolic pathways, cell proliferation, anchorage-independent growth, mitochondrial respiration, glycolytic function, and ATP production rates in those cells. Lastly, we performed a drug screening to identify agents specifically targeting the SDHA overexpressing tumor cells. We showed that SDHA overexpressing cells are characterized by enhanced energy metabolism, relying on both glycolysis and oxidative phosphorylation to meet their energy needs. In addition, SDHA-high phenotype was associated with cell vulnerability to glucose and glutamine deprivation, which led to a substantial reduction of ATP yield. We also identified an anti-metabolic compound shikonin with a potent efficacy against SDHA overexpressing ovarian cancer cells. Our data underline the unappreciated role of SDHA in reprogramming of ovarian cancer metabolism, which represents a new opportunity for therapeutic intervention.

## 1. Introduction

The reprogramming of cellular metabolism is a hallmark of cancer allowing tumor cells to survive and proliferate. The Warburg effect is an established signature of cancer metabolism and refers to the observation that tumor cells preferentially use glucose via glycolysis pathway rather than oxidative phosphorylation (OXPHOS) in mitochondria for energy production [1]. The reliance on glycolysis in cancer cells has been previously attributed to dysfunctional mitochondria [2,3]. However, recent data revealed that many tumor types have functional mitochondria and are not solely dependent on glycolysis for their energy requirements but also derive energy from OXPHOS [4,5,6]. For instance, subsets of ovarian cancers with metastatic phenotype or cancer stem cell-like features have been shown to be highly metabolically active with increased mitochondrial respiration [7,8,9]. A better understanding of tumor bioenergetics can reveal metabolic dependencies of cancer cells and facilitate development of new therapies.

To gain a greater insight into the ovarian cancer metabolism, we interrogated metabolic phenotypes of patient-derived xenografts (PDXs) developed from patients’ ovarian tumors [10]. We used proteomic approaches and discovered that the overexpressed subunit A of succinate dehydrogenase enzyme (SDHA) is associated with elevated mitochondrial metabolism in ovarian PDX models. The genomic data demonstrated that the *SDHA* gene amplification or overexpression is highly prevalent in ovarian carcinoma patients (19% of all cases) compared to other tumor types, which indicates its potential importance in reprogramming of ovarian cancer metabolism.

Succinate dehydrogenase (SDH) also known as mitochondrial complex II, couples oxidation of succinate to fumarate with reduction of ubiquinone to ubiquinol, thus directly connecting the mitochondrial electron transport chain (ETC) with the tricarboxylic acid (TCA) cycle [11]. SDH consists of four subunits, SDHA flavoprotein and SDHB iron-sulfur protein that function as the catalytic core, which is bound to SDHC and SDHD proteins that anchor the whole protein complex to the inner mitochondrial membrane [12]. It has been shown that a correct assembly of succinate dehydrogenase complex is critical for its function [13]. For instance, genetic mutation of SDH subunits results in severe assembly defects leading to a number of clinical pathologies including neurodevelopmental disorders or some rare malignancies [11,14,15,16,17]. Previous studies demonstrated that the mechanism of tumor development in patients with succinate dehydrogenase deficiency is associated with succinate accumulation. An excess amount of succinate inhibits HIF1α prolyl hydroxylase activity leading to HIF-1α stabilization and increased transcription of HIF1α regulated genes promoting tumorigenesis [11]. In addition, defective complex II assembly often results in accumulation of a free SDHA flavoprotein in the mitochondrial matrix [18]. While mutations of SDH subunits are very rare in ovarian cancer, the amplification or overexpression of a SDHA subunit is highly prevalent in this tumor type (~20% tumors). However, the role of SDHA upregulation and its impact on ovarian cancer metabolism has never been investigated, emphasizing the need for further research.

In this work, using various in vitro and in vivo ovarian cancer models, we investigated the biological consequences of SDHA overexpression and its impact on tumor phenotype. Our findings revealed that SDHA overexpression is associated with the improved ability of cells to survive and generate colonies in anchorage-independent conditions. This is an important feature of metastatic ovarian tumor cells that are able to survive and spread in peritoneal fluid (ascites) within the abdominal cavity. Further, we showed that the overexpression of SDHA enhanced ovarian cancer metabolism reflected as increased mitochondrial respiration and ATP production rate. Lastly, we performed a drug screening and identified an anti-metabolic compound shikonin known to disrupt glycolysis and amino acid metabolism [19,20]. We showed that shikonin exhibited a profound anti-tumor efficacy and selectivity towards SDHA overexpressing tumor cells in vitro superior to that observed with traditional chemotherapy.

Altogether, our study demonstrated that SDHA upregulation frequently occurs in ovarian cancer and contributes to reprogramming of energy metabolism towards highly metabolically active phenotype. Importantly, we showed here that SDHA overexpressing tumor cells can be effectively targetable by anti-metabolic compounds such as shikonin, which represents a new opportunity for therapeutic intervention in ovarian cancer.

## 2. Materials and Methods

### 2.1. Source of Ovarian Cancer Cells and PDX Tumor Models

The OVCAR3 cell line was purchased from ATCC (Manassas, VA, USA). The OVCAR4 cell line was a gift from Dr. Jones [21]. Caov3, TYKnu, and OVSAHO cell lines were purchased from JCRB Cell Bank (Osaka, Japan). OVCAR3 and OVCAR4 cell lines were authenticated by short tandem repeat (STR) profiling by ATCC. To ensure the identity and validity of our cell lines and to prevent potential problems associated with cell culture, such as cell line misidentification, contamination and genetic drift, we purchase cell lines form validated, reliable source and cryopreserve 20 vials of each cell line at low passage (passage 1–3). The cell line vials are kept protected in a lab cell line bank and are distributed to lab members according to the experimental needs. All cell lines were maintained in RPMI 1640 Medium containing L-glutamine 300 mg/L and D-glucose 2 g/L (#11875, Thermo Fisher Scientific, Waltham, MA, USA) supplemented with 10% fetal bovine serum (FBS) (#F0926, Sigma-Aldrich, St. Louis, MO, USA), in a standard humidified incubator at 37 °C in 5% CO_2_ and 95% O_2_ atmosphere. Cell lines were tested for Mycoplasma by Idexx BioAnalytics and were found negative for any contamination. Human fallopian tube tissues and established PDX tumor models were obtained from the PDX-PCT core facility at OMRF [10].

### 2.2. Animal Experiments

All animal procedures were approved by the OMRF’s Institutional Animal Care and Use Committee. For in vivo experiments, 6 week-old female NOD/scid mice (#1303, Jackson Laboratory, Bar Harbor, ME, USA) were implanted subcutaneously into the flank with tumor cells (5 × 10^6^ OVCAR4-SDHAi cells or 5 × 10^6^ OVCAR3-SDHAi cells). Mice with established subcutaneous tumors of ~100 mm^3^ volume were randomized and fed with doxycycline-supplemented food (pellets containing 200 mg/kg doxycycline, #S3888, Bio-Serv, Flemington, NJ, USA) or respective control diet (#S3888, Bio-Serv, Flemington, NJ, USA). Subcutaneous tumor volumes were calculated using the formula ½ (Length × Width^2^). Mice were monitored weekly for body weight, development and progression of ovarian tumors, and any symptoms of physical distress or illness. At indicated time points tumors were harvested from mice and assessed for SDHA expression levels. At the endpoint, animals were humanely euthanized by CO_2_ inhalation as described in the IACUC-approved animal use protocol (#22-01).

### 2.3. Reverse Phase Protein Array

RPPA experiments were performed in accordance with instructions from the RPPA Core facility at the MD Anderson Cancer Center, University of Texas. We prepared cell pellets containing 2 × 10^6^ of cells derived from 9 HGSOC PDXs that were sent to the RPPA core facility. Our PDX samples were analyzed for 367 validated targets. Linear values of normalized RPPA data were used to determine protein expression.

### 2.4. In Silico Analysis of TCGA Datasets

The current study used publicly available genomic datasets generated by the TCGA and processed these data in accordance with the TCGA publication guidelines [22]. The cBioPortal online analytical tool (http://www.cbioportal.org/public-portal/, accessed on 15 January 2020) was used to analyze TCGA data matrix [23]. To assess genomic alterations of metabolic genes, we used an ovarian cancer dataset that encompassed 617 ovarian serous cystadenocarcinoma specimens in the “Firehose Legacy” category [24]. We queried tumors with copy-number variance (CNV) and mRNA expression data presented by z-scores detected with U133 microarray only, which included 606 samples. To determine the occurrence of SDHA amplification in ovarian cancer vs. other tumors, we compared the SDHA amplification frequency in 25 studies representing diverse malignancies, which included 11,459 samples. The results were visualized in OncoPrint.

### 2.5. Quantitative Real-Time PCR

Total RNA was extracted with the Qiagen RNeasy Mini kit (Qiagen, Valencia, CA, USA), according to the Manufacturer’s protocol. cDNA was synthesized using iScript cDNA synthesis kit (#1708840, Bio-Rad, Hercules, CA, USA) following the manufacturer’s instructions. A 20 μL reaction volume was prepared with 1 μg purified RNA. cDNA was diluted 1:20 in nuclease-free water and 1μL was analyzed in triplicates by real-time PCR using the LightCycler 96 System (Roche) and PowerUp™ SYBR^®^ Green Master Mix (#A25742, Thermo Fisher Scientific, Waltham, MA, USA), with 0.2 μM of each primer in a total volume of 25 μL reaction mixture. Primers were ordered from Integrated DNA Technologies, Inc. (IDT) (Coralville, IA, USA). Primers were based on published sequences or designed using Prime 3 software (Prime 3), which are listed as the following sequences: forward primer and reverse primer. Human SDHA: 5′ AGG CTT GCG AGC TGC ATT TG 3′, and 5′ AGC CCT TCA CGG TGT CGT AG 3′; human β-actin (ACTB): 5′ TGA CCC AGA TCA TGT TTG AGA CC 3′, and 5′ GTC CAG ACG CAG GAT GGC ATT 3′.

### 2.6. WES (ProteinSimple)

Cells or tumors were homogenized and lysed in Buffer B (25 mM Tris-HCl, pH 7.5, 0.42 M NaCl, 1.5 mM MgCl_2_, 0.5 mM EDTA, 1 mM DTT, 25% sucrose, 1 mM Na_3_VO_4_, and 1× protease inhibitor cocktail) on ice for 15 min, followed by centrifugal clearing at 4 °C for 10 min at 10,000 rpm to recover whole cell lysates. For analysis of proteins using a capillary electrophoresis-based protein analysis system (WES; ProteinSimple, San Jose, CA, USA), cellular proteins (0.5 mg/mL) were separated and visualized using the standard instrument protocol. Primary antibodies used were: GAPDH (1:300, #sc-25778) from Santa Cruz Biotechnology (Santa Cruz, CA, USA); α-Tubulin (1:25, #2144S); SDHA (1:25, #11998S) from Cell Signaling Technology (Danvers, MA, USA). Anti-rabbit secondary antibodies were included in the Wes-Rabbit (12-230 kDa) Master Kit (#PS-MK14, ProteinSimple, San Jose, CA, USA).

### 2.7. Succinate Dehydrogenase Activity

The activity of succinate dehydrogenase was assessed by the Succinate Dehydrogenase Activity Colorimetric Assay (#K660-100, BioVision, Waltham, MA, USA). Ovarian cancer cell lines were stimulated with 100 ng/mL dox for 24 h to induce SDHA overexpression and their corresponding control cells were harvested and assayed immediately for SDH activity following the manufacturer’s instructions. The reaction was initiated by adding a blue colored artificial probe to accept electrons from the succinate to fumarate oxidation. Absorbance at 600 nm was recorded in kinetic mode at RT. The decrease in absorbance per unit time (slope) was compared to a standard curve obtained with known protein concentrations and reported as a relative fold change of SDH activity between examined cell lines [25].

### 2.8. Fumarate Levels

Fumarate levels were assessed by the Fumarate Assay Kit (cat. no. MAK060, Sigma-Aldrich, St. Louis, MO, USA). Ovarian cancer cell lines were stimulated with 100 ng/mL dox for 24 h to induce SDHA overexpression and their corresponding control cells were harvested and assayed immediately for fumarate levels following the manufacturer’s instructions. Briefly, 1 mM fumarate standard solution was serially diluted and plated into a 96-well plate, generating fumarate standard curve. Fumarate assay buffer was then added to each well to bring the total volume up to 50 μL. Ovarian cancer cells were harvested (10^6^ cells per well in triplicates), washed with cold PBS, then resuspended and homogenized with 100 μL of ice cold Fumarate Assay Buffer, and centrifuged 10,000× *g* for 5 min to remove precipitate. Next, 50 μL of sample and 100 μL of an appropriate master reaction mix was added to each well and incubated for 30 min at 37 °C protected from light. Absorbance was then measured at 450 nm using a microplate reader. To measure fumarate levels, a standard curve was prepared in a 96-well plate using fumarate standard solution. Concentration (mM) of fumarate in ovarian cancer samples was calculated as follows: Fumarate = (Sa/Sv) × D; where Sa is an amount of fumarate from standard curve (mM), Sv is a sample volume added into each well (μL), and D represents sample dilution factor.

### 2.9. Generation of Lentiviruses and Cell Transduction

To generate ovarian cancer cell lines conditionally overexpressing SDHA, we used cell lines with low SDHA expression and conditionally overexpress the SDHA coding sequence by transducing the cells with pLentiTRE/rtTA-SDHA lentivirus. To construct the pLentiTRE/rtTA-SDHA lentivirus, the coding sequence of SDHA gene was amplified by PCR from human ovarian cancer cells and flanked by restriction sites for the HpaI and PacI enzymes. Next, the sequence was subcloned into HpaI and PacI restriction sites of the tetracycline inducible (Tet-On) lentiviral expression vector pLentiTRE/rtTA carrying both TRE and rtTA cassettes. The SDHA presence was verified by sequencing. Recombinant lentiviruses were produced in HEK293T cells according to our standard protocols [26]. Ovarian cancer cell lines were then infected with lentivirus containing the tetracycline-inducible SDHA, followed by selection with blasticidin. The SDHA expression was induced by the addition of 100 ng/mL of doxycycline (dox) for 24 h. To knockdown SDHA gene, we used validated shRNA clone TRCN0000028085 from Sigma-Aldrich. The shRNA was cloned into pLKO.1-puro lentiviral vector (Sigma-Aldrich, St. Louis, MO, USA). Recombinant lentiviruses were produced in HEK293T cells according to standard protocols [26]. Ovarian cancer cell lines endogenously overexpressing SDHA were then infected with lentiviruses containing the shRNA against SDHA, or control shRNA with a scrambled sequence, followed by selection with puromycin, as described [26].

### 2.10. 3T5 Cell Proliferation Assay

The 3T5 cell proliferation assay was performed by plating 5 × 10^5^ cells per 10 cm tissue culture plate (each cell line was set up in triplicate), followed by counting and re-plating at the same density every 3 days for 13 days. Population doubling time was calculated using the formula ln(post-3-day cell count/5 × 10^5^)/ln(2). The given population doubling time was added to the cumulative doubling time of the previous count.

### 2.11. Soft Agar Colony Formation Assay

Soft agar colony formation assay was performed as previously published [27]. Briefly, cells were cultured in 6-well plates (4 × 10^4^ cells per well) in a mixture of 0.6% DifcoTM Noble Agar (#214220, BD Biosciences, Franklin Lakes, NJ, USA) and their respective media, which was added on top of a layer of 1% noble agar in culture medium. To induce SDHA overexpression, fresh doxycycline solution in culture medium (100 ng/mL final concentration) was added every two days to respective wells. Control cells received the same volume of culture medium. Cells were cultured for 10 days and then stained overnight with 200 μL of nitroblue tetrazolium chloride (#VWR0329, VWR Chemicals, Radnor, PA, USA). The visible colonies were photographed using a Leica 205 FCA microscope. Colony number and was estimated using Image J, version 1.52a.

### 2.12. Metabolic Flux Analysis

The Seahorse XFe24 Extracellular Flux Analyzer (Agilent Technologies, Santa Clara, CA, USA) was used to assess bioenergetic profiles of ovarian cancer cell lines [28]. To run seahorse assay, cells were evenly seeded (60,000 cells/well; following optimization of cell seeding number) into the XF24 cell culture plate (#102340-100, Seahorse XFe24 FluxPaks, Agilent Technologies, Santa Clara, CA, USA) and incubated for 24 h at 37 °C and 5% CO_2_. After 24 h of incubation, cell culture medium was replaced with Seahorse XF Base Medium (#103335-100, Agilent Technologies, Santa Clara, CA, USA) supplemented with 2 mM L-Glutamine (#G7513-100ML, Sigma-Aldrich, St. Louis, MO, USA), 1 mM Sodium Pyruvate (#13-115E, Lonza Bioscience, Basel, Switzerland), 10 mM Glucose (#G8270-100G, Sigma-Aldrich, St. Louis, MO, USA) with pH adjusted to 7.4. Then, the cells were placed in a non-CO_2_ incubator for 1 h at 37 °C required for cells to reach an optimal pH and temperature conditions prior to the start of the experiment. Following 1 h incubation, the XF24 cell culture plate was loaded into the Seahorse instrument, which measured OCR/ECAR at intervals of approximately 5–8 min. Depending on the seahorse assay, various pharmacological compounds interrupting mitochondrial respiration were injected via ports to determine their effects on mitochondria function. The plate included also control blank wells containing only media to which various reagents were added similar to experimental wells. The blanks were automatically subtracted from experimental wells by the instrument software. Three measurements of OCR/ECAR were obtained following injection of each compound modulating cellular respiration. Compounds used in Seahorse assays included 1 uM Oligomycin A (ATP synthase inhibitor, #495455-10MG, MilliporeSigma, Burlington, MA, USA), 1 uM of FCCP (protonophoric uncoupler, #C2920-10MG, MilliporeSigma, Burlington, MA, USA) and 1 uM of Antimycin A (complex III inhibitor, #A8674-25MG, MilliporeSigma, Burlington, MA, USA), 10 mM glucose (#G8270-100G, Sigma-Aldrich, St. Louis, MO, USA), 50 mM 2-deoxy-D-Glucose (#14325, Cayman Chemical, Ann Arbor, MI, USA). Compound concentrations were optimized prior to experiments. The measurements were normalized with cell number and total protein levels (Bradford protein assay).

### 2.13. Seahorse XF Real-Time ATP Rate Assay Calculations

We performed calculations (validated by Agilent [29]) based on known reaction stoichiometry, where OCR, ECAR, and PER data were converted to mitochondrial or glycolytic ATP production rates. The calculated parameters included: mitochondrial OCR (pmol O_2_/min) = basal OCR—non-mitochondrial OCR after antimycin A (AA) injection; mitochondrial PER (mitoPER, pmol H^+^/min) = CO_2_ conversion factor × mitochondrial OCR, in which the CO_2_ conversion factor for XFe24 well plates is defined as 0.60 [30]; basal glycolysis (glycoPER, pmol H^+^/min) = total PER − mitochondrial PER; glycoATP Production Rate (pmol ATP/min) = glycoPER; OCR_ATP_ (pmol O_2_/min) = basal OCR basal—OCR after oligomycin injection; mito ATP Production Rate (pmol ATP/min) = OCRATP × 2 (pmol O/pmol O_2_) × P/O (pmol ATP/pmol O), where P/O value of 2.75 derives from the stoichiometry of reaction representing the number of molecules of ATP synthesized per atom of O reduced by an electron pair; and total ATP Production Rate (pmol ATP/min) = glycol ATP Production Rate + mito ATP Production Rate [31].

### 2.14. Glycolytic Rate Assay Calculations

We performed the Glycolytic Rate Assay, which measures a Proton Efflux Rate (PER). The obtained PER values allowed to calculate determinants of glycolysis: mitochondrial PER (mitoPER) = CO_2_ conversion factor × (OCR − non-mitochondrial OCR after antimycin A injection), in which the CO_2_ conversion factor for XFe24 well plates is defined as 0.60 [30]; basal glycolysis (glycoPER) = (total PER -2-DG PER, which includes other sources of extracellular acidification that are not attributed to glycolysis or mitochondrial TCA activity)− mitochondrial PER; maximal glycolysis = PER after Antimycin A injection—2-DG PER (post-2-DG acidification); and glycolytic reserve = Maximal glycolysis—glycoPER.

### 2.15. Nutrient Deprivation Assay

For glucose deprivation conditions, respective cells were cultured in glucose free RPMI 1640 medium (Gibco #11879020), supplemented with 1 mM sodium pyruvate and low concentration of D-glucose (200 mg/L). Glutamine deficient medium was prepared using glutamine free RPMI 1640 medium (Gibco #21870076) supplemented with 1 mM sodium pyruvate and low concentration of glutamine (30 mg/L). To inhibit glycolysis, galactose medium was generated using glucose free RPMI 1640 medium (Gibco #11879020), supplemented with 1 mM sodium pyruvate and optimal concentration of galactose (2 g/L). The cells were cultured in complete medium or nutrient deprived medium for 48 h prior metabolic flux analysis (as previously described [32]).

### 2.16. Anti-Metabolic Compound Library and Drug Screening Assay

A customized anti-metabolic compound library was generated from Selleck Chemicals inventory (#L3000, #L3700, #L5700, and #L6900), and consisted of 64 selective agents targeting various aspects of cellular metabolism (Supplementary Table S2). The in vitro drug screening assay was carried out by treating exponentially growing ovarian cancer cells with 64 individual anti-metabolic agents followed by an MTT assay to measure cell viability. The cell viability was assessed by the Quick Cell Proliferation Assay kit II (BioVision, Milpitas, CA, USA). Briefly, cells were seeded in a 96-well plate in 100 μL of their respective media at a density of 2000–5000 cells per well. After cells were cultured for 24 h, the medium was aspirated and cells were exposed to desired drug concentration(s) for 4 days. In addition, a vehicle control corresponding to the highest DMSO concentration, which did not exceed 0.2%, was also included. Next, cells were incubated with the WST reagent for 2 h and absorbance was determined at 450 nm. Absorbance measurements were normalized to the DMSO control wells. Normalized values were plotted as an average ± SD of three wells per condition and these data were analyzed to determine the changes in cell viability.

### 2.17. Statistical Analysis

All in vitro experiments were performed three times and in triplicate when applicable. Values are presented as mean ± SD, or as mean ± SEM. Statistical analysis of in vitro assays or in vivo data was done using unpaired *t*-test, multiple *t*-test, or analysis of variance (ANOVA) followed by Tukey’s multiple comparisons test whenever applicable. *p* < 0.05 was considered significant. Statistical analysis was performed using GraphPad Prism 6.0 Software (San Diego, CA, USA).

## 3. Results

### 3.1. High Frequency of SDHA Amplification and Upregulation in Ovarian Cancer

**Figure 1:**
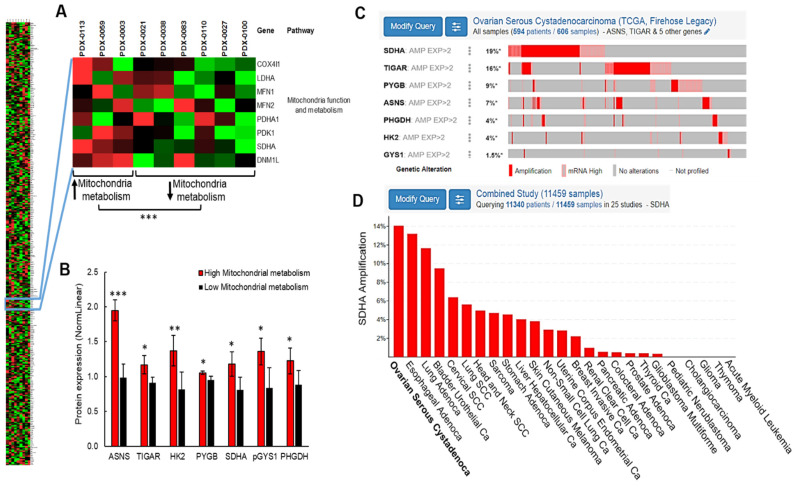
High frequency of SDHA amplification and upregulation in ovarian cancer. (**A**) A heatmap representing the expression of 367 proteins in 9 HGSOC PDX models. Two PDX subsets were identified, one with increased, and the other with decreased expression of proteins associated with mitochondrial function (unpaired *t*-test). (**B**) The analysis of multiple metabolic pathways in PDX with high vs. low mitochondrial metabolism revealed 7 differentially expressed proteins represented by gene ID. Data are represented as mean ± SD; * = *p* < 0.05, ** = *p* < 0.01, and *** = *p* < 0.001 (multiple *t*-test). (**C**) In silico TGCA data analysis revealed that amongst 7 differentially expressed proteins between PDX with high vs. low mitochondrial metabolism, SDHA amplification and upregulation was the predominant genetic alteration reaching 19% of HGSOC cases. (**D**) The SDHA amplification occurs with the highest frequency in ovarian carcinoma when compared with 25 other malignancies. For additional information see Supplementary Table S1 and Figure S1.

To gain a better understanding of ovarian cancer metabolism, we utilized PDX models [10] derived from tumors of patients diagnosed with high-grade serous ovarian carcinoma (HGSOC), and performed an analysis of metabolic pathways by Reverse Phase Protein Array (RPPA). Since it has been shown that ovarian cancer cells with aggressive phenotype show enhanced metabolism and increased oxidative phosphorylation in mitochondria [7,8,9], we interrogated metabolic phenotypes of ovarian PDXs focusing on mitochondria function and metabolism. The quantification of protein expression by RPPA revealed a subset of PDX models with phenotype consistent with elevated mitochondrial metabolism (PDX-0003, PDX-0059 and PDX-0113), while the phenotype of remaining models was consistent with reduced mitochondria function (PDX-0100, PDX-0110, PDX-0038, PDX0027, PDX-0021 and PDX-0083) (Figure 1A and Supplementary Table S1). Next, we compared the protein content within selected metabolic pathways (including amino acids metabolism, glycogen metabolism, glycolysis, lipid metabolism, and mitochondria function and metabolism) between PDX with increased vs. decreased mitochondrial metabolism (Figure 1A and Supplementary Table S1). This analysis led to the identification of 7 differentially expressed proteins (SDHA, ASNS, TIGAR, HK2, PYGB, GYS1, and PHGDH represented by gene name), which expression was significantly changed between PDXs with high vs. low mitochondrial metabolism (Figure 1B).

Further, we performed in silico analysis of the The Cancer Genome Atlas (TCGA) dataset encompassing 617 ovarian serous cystadenocarcinoma specimens [24]. We interrogated the frequency of molecular alterations of genes encoding the 7 differentially expressed proteins to identify the potential regulators of cellular metabolism in ovarian cancer. This analysis revealed that the most frequently altered metabolic gene in HGSOC patients is *SDHA* that encodes a subunit A of succinate dehydrogenase enzyme. The *SDHA* amplification or overexpression occurs in 19% of HGSOC cases, which constitutes one fifth of the patient population (Figure 1C). We observed no correlation between *SDHA* amplification/overexpression status and disease free survival. However, there was a tendency towards improved overall survival of ovarian cancer patients with *SDHA* amplification/overexpression (Supplementary Figure S1). Next, we analyzed the frequency of *SDHA* amplification across different tumor types, and found that ovarian cancer shows the highest *SDHA* amplification rates out of the 25 diverse malignancies (Figure 1D). These findings indicate that SDHA upregulation could play an essential role in a reprogramming of cellular metabolism contributing to ovarian cancer pathogenesis.

### 3.2. Succinate Dehydrogenase Expression and Activity in High-Grade Serous Ovarian Cancer Models

**Figure 2:**
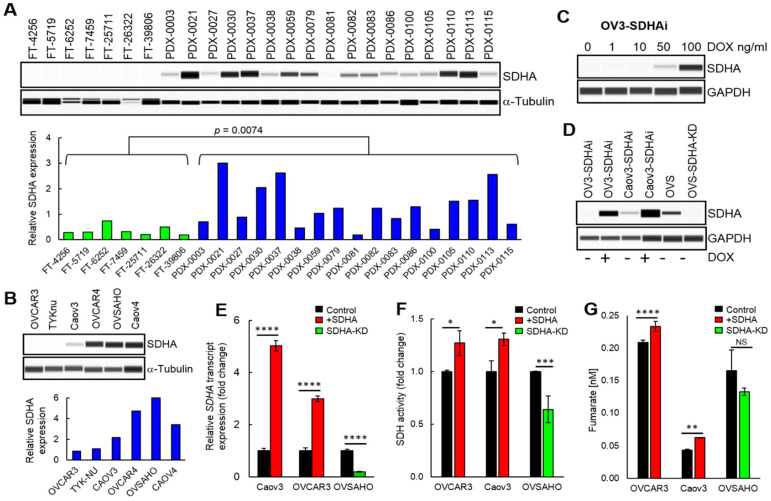
Evaluation of SDHA expression in ovarian cancer. (**A**) WES analysis of fallopian tubes “FT” or HGSOC PDX assayed for SDHA and α-Tubulin. Graph represents normalized quantification of SDHA protein expression using Compass for Simple Western Software. HGSOC PDXs showed significantly higher expression of SDHA than healthy FTs (Unpaired *t*-test). (**B**) WES analysis of ovarian cancer cell lines assayed for SDHA and αTubulin. (**C**) Dose-dependent induction of SDHA by dox treatment for 24 h. (**D**) SDHA levels in cell lines conditionally overexpressing SDHA (SDHAi) following incubation with 100 ng/mL dox for 24 h. (**E**) The qRT-PCR analysis of SDHA transcript levels following induction of SDHA expression or SDHA knockdown (KD). Graph represents relative fold change in SDHA expression (normalized with β-actin expression). (**F**) Assessment of succinate dehydrogenase activity in ovarian cancer cell lines following SDHA induction or SDHA KD. Relative fold change in succinate dehydrogenase activity relative to control. (**G**) Levels of intracellular fumarate following SDHA induction or SDHA KD in ovarian cancer cell lines. Data are represented as mean ± SD, (unpaired *t*-test, **** = *p* < 0.0001 *** = *p* < 0.001 ** = *p* < 0.01 * = *p* < 0.05). The uncropped blots are shown in Figure S6.

Currently, very limited data exist regarding the succinate dehydrogenase levels and function in ovarian cancer or other tumor types. In this work, we quantified SDHA levels in fallopian tubes from patients (since HGSOC subtype originates from this tissue [33]) and PDX models derived directly from patient’s HGSOC tumors [10]. WES (capillary-based immunoassay system from ProteinSimple) analysis revealed that SDHA expression is significantly lower in fallopian tubes than HGSOC PDXs (Figure 2A). The PDX models showed a range of SDHA expressions. We identified PDX lines with high expression of SDHA (PDX-0021, PDX-0030, PDX-0037, and PDX-0113), where SDHA protein levels are greater than the 75th percentile value of SDHA levels in remaining PDXs. The SDHA-low PDXs (below 25th percentile) included PDX lines such as PDX-0038, PDX-0081, PDX-0110, and PDX-0115 (Figure 2A). Further, we evaluated endogenous levels of SDHA in ovarian cancer cell lines by WES. We identified SDHA-high cell lines (OVCAR4, OVSAHO, and Caov4) and those with low SDHA levels (OVCAR3, TYKnu, and Caov3) (Figure 2B). The SDHA-high cell lines demonstrated 2–3 fold higher SDHA protein expression than the SDHA-low cell lines.

To study the functional consequences of SDHA upregulation in ovarian cancer, we generated a panel of ovarian cancer cell lines, where we conditionally overexpressed SDHA in SDHA-low cell lines using doxycycline (dox) inducible system (Figure 2C,D), or performed stable SDHA knockdown (KD) in OVSAHO cells (Figure 2D). Treatment with 100 ng/mL of dox for 24 h was sufficient to induce SDHA upregulation in respective cell lines (Figure 2D). Furthermore, the analysis of SDHA mRNA levels following SDHA induction by dox treatment or SDHA knockdown revealed 3–5 fold changes in transcript expression, which reflected the respective changes in SDHA protein levels (Figure 2E).

We assessed an enzymatic activity of succinate dehydrogenase in our ovarian cancer cell lines conditionally overexpressing SDHA or SDHA KD cells using Succinate Dehydrogenase Activity Assay. SDHA overexpression was induced by treating cells with 100 ng/mL of dox for 24 h prior the assay. The results demonstrated that the overexpression of SDHA in OVCAR3 and Caov3 cell lines resulted in a significant increase in succinate dehydrogenase activity. In contrast, the SDHA KD resulted in a substantial suppression of the enzyme activity in OVSAHO cells (Figure 2F). Succinate dehydrogenase couples oxidation of succinate to fumarate, thus an increase in the enzyme activity is expected to result in fumarate accumulation. As predicted, the overexpression of SDHA in ovarian cancer cell lines was associated with increased fumarate levels (as assessed by Fumarate Assay, Figure 2G). Together, these data validated a proper enzymatic function of exogenously overexpressed SDHA in ovarian cancer cell lines.

### 3.3. The Effect of SDHA Overexpression on Cell Proliferation and Anchorage-Independent Growth

**Figure 3:**
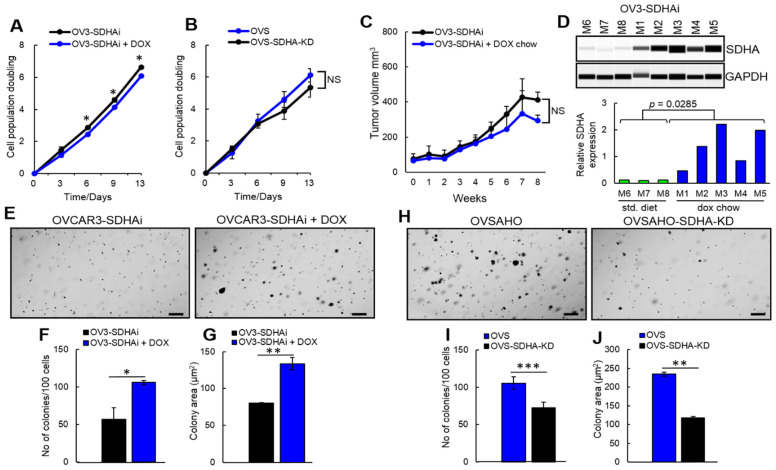
The effect of SDHA overexpression on cell proliferation and anchorage-independent growth. (**A**) The effect of inducible SDHA overexpression on OVCAR3 cell proliferation was assessed by 3T5 cell doubling assay. (**B**) The effect of SDHA KD on proliferation of OVSAHO cells. (**C**) In vivo OVCAR3-SDHAi subcutaneous tumor growth rate in NOD/scid mice. The SDHA over expression in tumors was induced by feeding mice with dox chow. Control mice were fed a control diet. (**D**) Quantification of the SDHA expression by WES in tumors resected from mice shown in (**C**). (**E**) Images represent anchorage-independent growth and colony formation of OVCAR3-SDHAi cells stimulated with dox. Number of colonies in cells overexpressing SDHA vs. controls was quantified in (**F**) and their size was assessed in (**G**). (**H**) Anchorage-independent growth of OVSAHO-SDHA-KD vs. control cells. Number of colonies in cells depleted from SDHA vs. controls was quantified in (**I**) and their size was assessed in (**J**). Data are expressed as mean ± SEM. Statistical significance of data was assessed by multiple *t*-test (3T5 assay) and unpaired *t*-test (WES and anchorage-independent growth assay); *** = *p* < 0.001 ** = *p* < 0.01 * = *p* < 0.05). The uncropped blots are shown in Figure S6.

To evaluate if SDHA overexpression affects cell proliferation, we measured the cumulative population doubling of cells with inducible SDHA overexpression (SDHAi) or those with SDHA KD. Some cell lines such as OVCAR3-SDHAi showed a significant reduction of cell proliferation rate, while others (Caov3-SDHAi and OVSAHO) showed no effect of SDHA on cell proliferation (Figure 3A,B and Figure S2A). Collectively, our findings demonstrated a tendency to reduced ovarian cancer cell proliferation following SDHA overexpression in vitro.

Further, to determine the effect of SDHA overexpression on tumor growth in vivo, we subcutaneously implanted 5 × 10^6^ of SDHA dox-inducible ovarian cancer cells into the flank of NOD/scid mice. When tumors reached 100 mm^3^ the animals were randomized into two experimental groups: (a) control group fed with standard diet, and (b) SDHA overexpression group fed with dox-containing chow (Figure 3C,D). Tumor volume was measured on a weekly basis for 8 weeks following mice randomization. Tumors from each group were assessed for SDHA expression by WES to confirm SDHA overexpression phenotype in mice fed with dox chow vs. standard diet. We observed a tendency towards reduced tumor growth rate in mice fed with dox chow when compared with mice fed with standard diet. (Figure 3C).

As another approach to test the ability of SDHA overexpressing cells to proliferate and form colonies, we evaluated colony formation and anchorage-independent growth of cells. We observed that SDHA overexpression was associated with both higher number and size of ovarian cancer cell colonies, while the SDHA KD reduced formation of cell colonies (Figure 3E–J, and Supplementary Figure S2B). These data indicate that while SDHA overexpressing cells tend to proliferate slower, these cells have improved ability to survive and generate colonies in anchorage-independent conditions.

### 3.4. Bioenergetic Profiles of Ovarian Cancer Cell Lines Endogenously Overexpressing SDHA

To determine the differences in bioenergetic profiles of ovarian cancer cell lines endogenously overexpressing SDHA and those characterized by naturally low SDHA expression levels, we performed the Seahorse XF Cell Mito Stress Test that measures key parameters of mitochondrial and glycolytic function [34]. The assay exposes cells to pharmacological modulators to obtain parameters such as basal, maximal, and non-mitochondrial respiration, as well as respiratory reserve capacity. We used 4 ovarian cancer cell lines and recorded 3 baseline oxygen consumption rate (OCR) measurements, then oligomycin A was added to inhibit ATP synthase suppressing ATP-linked respiration. Further, an addition of carbonyl cyanide-p-trifluoromethoxyphenylhydrazon (FCCP) resulted in inhibition of the electron transport chain resulting in the maximal respiration. The difference between maximal and basal respiration reveals respiratory reserve capacity that reflects the ability of cells to respond to increased energy demand or cellular stress. Lastly, the addition of antimycin A (complex III inhibitor) shuts down mitochondrial respiration [34].

**Figure 4:**
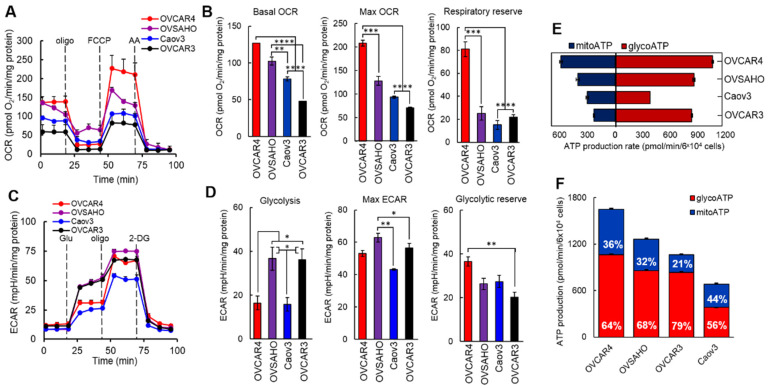
Bioenergetics profiles of ovarian cancer cell lines. (**A**) OCR was measured in ovarian cancer cell lines by the Seahorse XF Cell Mito Stress Test. Cells were challenged by oligomycin A “oligo”, FCCP, and antimycin A “AA”. (**B**) Basal OCR represents the mitochondrial respiration at resting state, the maximum respiration indicates the cellular response to an increased ATP demand, and respiratory reserve indicates the cell capacity to utilize mitochondria during periods of high-energy expenditure. Data are represented as mean ± SEM, illustrating differences in OCR between cell lines (unpaired *t*-test, **** = *p* < 0.0001, *** = *p* < 0.001, ** = *p* < 0.01). (**C**) ECAR was measured in ovarian cancer cell lines by the Seahorse XF Glycolysis Stress Test. Cells were challenged by glucose, oligomycin A, and 2-DG. (**D**) Increase in ECAR in response to glucose represents a rate of glycolysis under basal conditions, maximal ECAR represents the cellular maximum glycolytic capacity, and glycolytic reserve defines the capacity to increase glycolysis when mitochondria are compromised. Data are represented as mean ± SEM, illustrating differences in ECAR between cell lines (unpaired *t*-test, ** = *p* < 0.01 * = *p* < 0.05). (**E**) Seahorse XF Real-Time ATP Rate Assay was performed using ovarian cancer cell lines revealing OCR-linked (mitoATP) and ECAR-linked (glycoATP). Graph shows ATP production rates in basal conditions. (**F**) Graph represents a distribution of mitochondrial and glycolytic ATP production rates represented as % of mitoATP and % of glycoATP. All Seahorse experiments were performed for a minimum of 2 times in triplicates.

The results revealed that SDHA-high cell lines (OVCAR4 and OVSAHO) demonstrated higher basal OCR readings compared to SDHA-low cell lines (Caov3 and OVCAR3). All cell lines responded to FCCP by increasing their OCR to maximal rates, which were the highest in SDHA-high cell lines. The respiratory reserve capacity was also the highest in SDHA-high cell lines (Figure 4A,B).

Next, to assess the glycolytic function of SDHA-high vs. SDHA-low ovarian cancer cell lines, we performed the Seahorse XF Glycolysis Stress Test, which directly measures the extracellular acidification rate (ECAR) in live cells [35]. First, cell lines were incubated in glucose free medium allowing for 3 ECAR readings representing non-glycolytic acidi fication. Addition of glucose resulted in a rapid increase in ECAR revealing the rate of glycolysis under basal conditions. The injection of oligomycin A inhibited mitochondrial ATP generation shifting the energy production to glycolysis, which resulted in elevation of ECAR revealing the maximum glycolytic capacity of cells. Lastly, the addition of 2-Deoxy-D-Glucose (2-DG, a glucose analog), suppressed glycolysis resulting in a drop in ECAR. The difference between maximal glycolytic capacity and glycolysis rate defines glycolytic reserve.

The results demonstrated a lack of correlation between glycolysis rate and SDHA overexpression status (Figure 4C,D). For instance, the SDHA-high cell line OVCAR4 showed low glycolytic rate, while the other SDHA-high cell line OVSAHO showed high glycolytic rate. However, the SDHA-high cell lines tend to have higher maximal glycolytic capacity (max ECAR) and glycolytic reserve when compared with SDHA-low cell lines (Figure 4D).

### 3.5. ATP Production Rates in Ovarian Cancer Cell Lines Endogenously Overexpressing SDHA

The ATP production rate is a highly informative measurement in understanding cancer cell bioenergetics. Therefore, we quantified cellular ATP production rates in SDHA-high and SDHA–low ovarian cancer cell lines by performing Seahorse XF Real-Time ATP Rate Assay [29]. The assay uses metabolic modulators (oligomycin A and antimycin A) to measure total ATP production rates, and allows to distinguish between the mitochondrial ATP fraction and the glycolytic ATP fraction. We performed calculations based on known reaction stoichiometry, where OCR, ECAR, and PER data were converted to mitochondrial or glycolytic ATP production rates (see Materials and Methods, Section 2.13).

The results demonstrated that SDHA-high cell lines (OVCAR4 and OVSAHO) showed higher ATP production rates when compared with SDHA-low cell lines (Figure 4E,F). All cell lines regardless of SDHA status, demonstrated higher rates of ATP production from glycolysis, which suggests that ovarian cancer preferentially uses glycolysis pathway to fulfill the energy requirements (Figure 4F).

### 3.6. The Effect of Inducible SDHA Overexpression on Mitochondrial Respiration and Glycolysis

**Figure 5:**
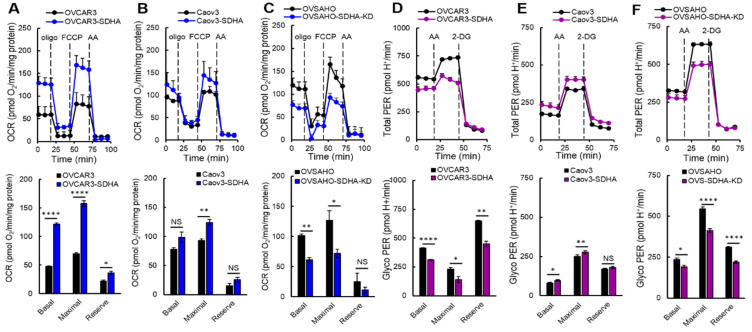
SDHA increases mitochondrial respiration in ovarian cancer cell lines. (**A**–**C**) OCR was measured in ovarian cancer cell lines overexpressing SDHA or SDHA KD cells vs. controls by the Seahorse XF Cell Mito Stress Test. Cells were challenged by oligomycin “oligo”, FCCP, and antimycin A “AA”. Basal OCR represents the mitochondrial respiration at resting state, the maximum respiration indicates the cellular response to an increased ATP demand, and respiratory reserve indicates the cell capacity to utilize mitochondria during periods of high-energy expenditure. (**D**–**F**) Glycolytic Rate Assay was performed to calculate Proton Efflux Rate (PER) derived from glycolysis (glycoPER) in ovarian cancer cell lines overexpressing SDHA or SDHA KD cells vs. controls. Cells were challenged with AA to inhibit mitochondrial oxygen consumption followed by addition of 2-DG. The obtained PER values allowed to calculate basal glycolysis and maximal glycolysis, and glycolytic reserve. Data are represented as mean ± SEM, illustrating differences in OCR between cell lines (unpaired *t*-test, **** = *p* < 0.0001, ** = *p* < 0.01 * = *p* < 0.05, NS = not significant).

Here, we evaluated the effect of a conditional overexpression of SDHA or stable SDHA KD in ovarian cancer cell lines on mitochondrial respiration and glycolysis. The Seahorse XF Cell Mito Stress Test revealed that the inducible SDHA overexpression significantly increased basal, maximal and reserve respiration in the OVCAR3 cell line (Figure 5A). We also detected an increase in these parameters in SDHA overexpressing Caov3 cells (Figure 5B). As expected, SDHA KD led to a significant inhibition of basal and maximal respiration and a suppression of the respiration reserve capacity in OVSAHO cell line (Figure 5C). These data are in agreement with those obtained with endogenous SDHA overexpression, and confirms that SDHA overexpression promotes mitochondrial respiration.

Next, we performed the Glycolytic Rate Assay, which measures a Proton Efflux Rate (PER). This method allows to calculate PER derived from glycolysis (glycoPER) discounting the effect of mitochondria CO_2_-dependent acidification. Therefore, glycoPER faithfully represents the lactate production rate from glycolysis [36]. First, we used pairs of ovarian cancer cell lines with and without SDHA overexpression and recorded basal rates of OCR, ECAR, and PER. Next, an inhibitor of mitochondrial ETC (antimycin A) was injected to inhibit mitochondrial oxygen consumption (and therefore CO_2_-derived protons). The following addition of 2-DG resulted in decrease in PER values providing qualitative confirmation that the PER produced prior to the injection is primarily due to glycolysis (Figure 5D–F). The obtained PER values allowed us to calculate determinants of glycolysis (see Materials and Methods, Section 2.14).

The results revealed that the effect of SDHA overexpression on glycolysis is different in individual cell lines. For instance, in the OVCAR3 cell line that is naturally highly glycolytic, the SDHA overexpression suppressed glycolysis (Figure 5D), while in Caov3 cell line that depends more on mitochondrial respiration, the SDHA overexpression increased glycolysis (Figure 5E).

**Figure 6:**
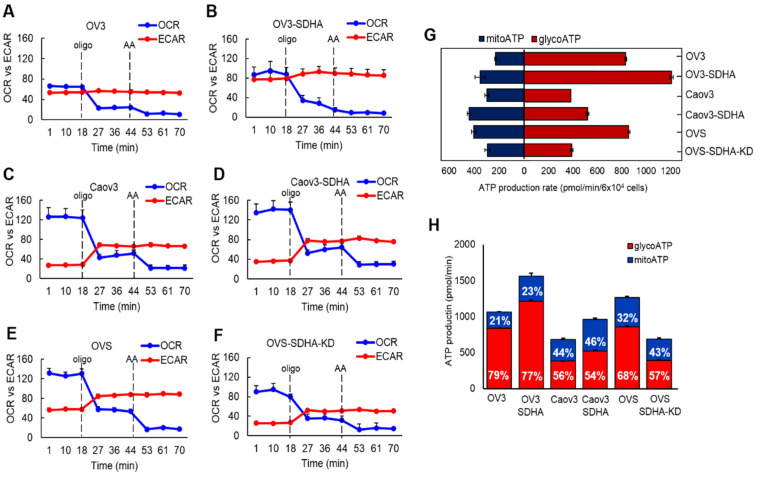
SDHA increases ATP production rate in ovarian cancer cell lines. (**A**–**F**) Seahorse XF Real- Time ATP Rate Assay was performed using ovarian cancer cell lines overexpressing SDHA or SDHA KD cells vs. controls. Both OCR and ECAR were simultaneously measured in live cells revealing OCR-linked (mitoATP) and ECAR-linked (glycoATP) in basal conditions, and after addition of oligomycin “oligo”, and antimycin A “AA”. (**G**) Quantitative measurement of ATP production rate from mitochondrial and glycolytic pathways. (**H**) Graph represents a distribution of mitochondrial and glycolytic ATP production rates shown as % of mitoATP and % of glycoATP.

Next, we used selected cell lines with conditional SDHA overexpression or stable SDHA KD and quantified ATP production rate by Seahorse XF Real-Time ATP Rate Assay (Figure 6A–H) [29]. We observed that the SDHA overexpression significantly increased the total ATP production rate, while SDHA KD reduced ATP yield (Figure 6G). Further analysis demonstrated that the increase in total ATP production rate is associated with increased ATP production from both mitochondria and glycolysis (we observed slightly higher ATP yield from mitochondria OXPHOS, Figure 6H).

In conclusion, this study demonstrated that the SDHA overexpression contributes to metabolic enhancement associated with significantly increased mitochondrial respiration as well as glycolysis in some of the cell lines leading to increased ATP production rate.

### 3.7. The Impact of SDHA Overexpression on Ovarian Cancer Dependence on Glucose or Glutamine

As another approach to assess the impact of SDHA upregulation on cellular metabolism, we tested ovarian cancer dependence on nutrients essential for tumor growth. We deprived ovarian cancer cells of selected metabolic nutrients such as glucose or glutamine, or replaced glucose with galactose in culture medium. Glucose is required by cells to produce ATP via glycolysis, and to fuel TCA cycle with pyruvate. Replacing glucose with galactose forces cells to rely almost exclusively on mitochondrial OXPHOS for ATP production [37]. Glutamine is an essential nutrient for cancer cells required to fuel TCA cycle with metabolic intermediates [38].

**Figure 7:**
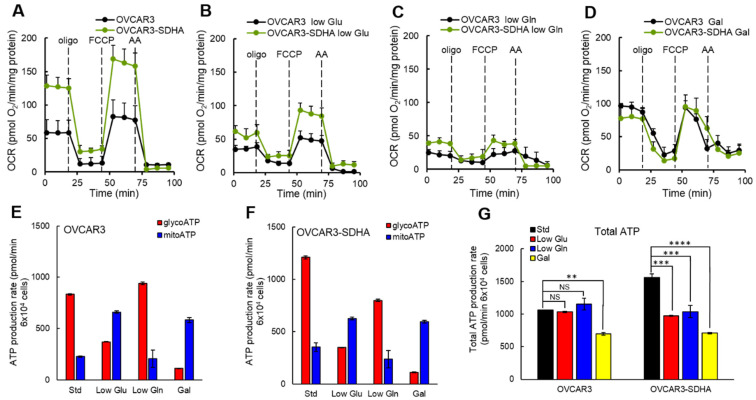
The effect of nutrient deprivation on cellular energetics of ovarian cancer cell lines overexpressing SDHA. (**A**) OCR was measured in OVCAR3 +/−SDHA cell lines in complete medium. SDHA overexpression led to a significant increase in basal, maximal and reserve respiration. (**B**) In low glucose (Glu) medium, the OCR was suppressed in both cell lines, when compared to OCR in complete medium. (**C**) In low glutamine (Gln) medium, the OCR was suppressed the strongest in both cell lines. (**D**) In galactose (Gal) medium, the basal respiration was higher than the basal respiration in low Glu or Gln medium reflecting an increased reliance on OXPHOS in cells with substantially inhibited glycolysis. (**E**,**F**) Quantitative measure of an ATP production rate from mitochondrial OXPHOS (mitoATP) or glycolysis (glycoATP) in various nutrient deprivation conditions in OVCAR3 (**E**) and OVCAR3-SDHA (**F**) cell lines. (**G**) Total ATP production rate in OVCAR3 cell lines exposed to nutrient deprivation. Data are represented as mean ± SEM (unpaired *t*-test, **** = *p* < 0.0001, *** = *p* < 0.001, ** = *p* < 0.01, NS = not significant).

To determine if SDHA overexpression redirects cellular metabolism in response to nutrient deprivation, we cultured selected ovarian cancer cell lines in low glucose, low glutamine or galactose medium (where glucose was replaced with galactose) and assessed cellular respiration by the Seahorse XF Cell Mito Stress Test (Figure 7A–D and Figure S3A–H). We observed that in a complete medium, SDHA overexpression significantly increased basal, maximal and reserve respiration in OVCAR3 cells (Figure 7A). In conditions with reduced glucose (Figure 7B), the mitochondrial respiration significantly decreased in both cell lines (OVCAR3 and OVCAR3-SDHA). Deprivation of these cells from glutamine resulted in even further suppression of mitochondria respiration than in low glucose medium (Figure 7C). In galactose medium, however, the basal mitochondrial respiration was higher than the basal respiration in conditions with reduced glucose or glutamine (Figure 7D), which is consistent with an increased reliance on oxidative phosphorylation (usually via glutamine driven metabolism) in cells with substantially inhibited glycolysis [39]. Similar findings were observed with Caov3 cell lines (Supplementary Figure S3A–H). Together, these data showed that a reduced supply of glucose or glutamine inhibited mitochondrial respiration to certain degree in ovarian cancer cells with and without SDHA overexpression.

To better understand cellular energetics in SDHA overexpressing ovarian cancer cells deprived of nutrients, we quantified ATP production rate. We observed that ovarian cancer cells showed substantial metabolic plasticity, which enabled the cells to switch between glycolysis and oxidative phosphorylation to adapt to changing nutrient conditions during experiment (Figure 7E,F and Supplementary Figure S3I–K). Our data revealed that in complete medium OVCAR3 cell lines produced more ATP from glycolysis than from mitochondrial respiration (Figure 7E,F). In low glucose medium or following glycolysis inhibition by galactose medium, OVCAR3 cell lines switched to mitochondrial OXPHOS pathway. This resulted in a significant decrease in ATP production from glycolysis that has been compensated with a substantially higher ATP yield from mitochondrial OXPHOS (Figure 7E,F). Deprivation of these cells from glutamine resulted in a high ATP yield from glycolysis and a suppression of ATP production via mitochondrial respiration.

Further, we analyzed a total ATP production rate in OVCAR3 cell lines in conditions with low glucose or low glutamine supply. Following glucose deprivation, OVCAR3 cells were capable to fully compensate for a loss in glycolytic ATP production by increasing their mitochondrial ATP production rate (Figure 7E). Such metabolic switch resulted in no difference in the total ATP production rate in OVCAR3 cells cultured in standard medium vs. those grown in glucose deprived medium (Figure 7G). Similarly, after deprivation of glutamine, OVCAR3 cells increased glycolytic flux and ATP yield from glycolysis pathway (Figure 7E), and a total ATP production rate remained the same in a low glutamine medium as in a standard medium (Figure 7G). In contrast, the SDHA overexpressing cells (OVCAR3-SDHA), were not able to fully compensate for the loss in ATP production following glutamine or glucose deprivation (Figure 7F,G). As a result, the cells demonstrated significantly reduced total ATP content in nutrient deprived medium vs. complete medium (Figure 7G). We also noted that a complete inhibition of glycolysis by galactose medium significantly reduced the total ATP production rate in both cell lines, with a stronger reduction of total ATP content in SDHA overexpressing cells (Figure 7G). Similar observations were made with Caov3 cell line, where a nutrient deprivation more profoundly reduced a total ATP yield in SDHA overexpressing cells than the respective control cells (Supplementary Figure S3I–K).

Collectively, these findings indicate that SDHA overexpressing cell lines are not able to fully compensate for the loss of ATP production following glucose or glutamine deprivation, which results in a significant drop in a total ATP yield. In contrast, the corresponding SDHA-low cells demonstrated an improved metabolic capacity allowing them to better maintain a constant ATP production rate in nutrient deprived conditions. These data also suggest that SDHA overexpressing cells can be more sensitive to a deficiency in essential nutrients or cellular stress.

### 3.8. Shikonin, a Potent Compound Specifically Targeting SDHA Overexpressing Ovarian Cancer Cells

**Figure 8:**
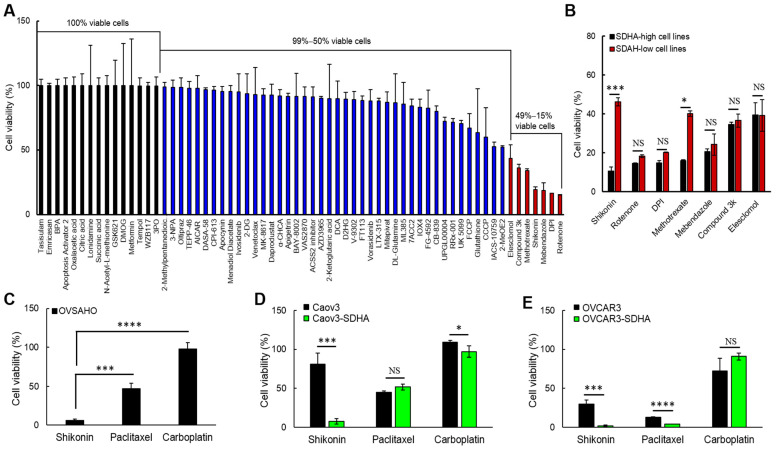
The SDHA overexpressing cell lines are highly sensitive to shikonin. (**A**) Ovarian cancer cell lines (OVCAR3, OVCAR4, TYKnu, and OVSAHO) were exposed to anti-metabolic compound library. The most potent agents are denoted in red. (**B**) The anti-cancer efficacy of the most potent compounds was evaluated in SDHA-high vs. SDHA-low cell lines. Shikonin was the most effective agent in specifically killing SDHA overexpressing cells. (**C**–**E**) Ovarian cancer cell lines endogenously overexpressing SDHA (OVSAHO) or conditionally overexpressing SDHA (Caov3-SDHA and OVCAR3-SDHA) were treated with shikonin or chemotherapy (paclitaxel, carboplatin) to assess % of viable cells. (**C**) Shikonin is significantly more effective in killing OVSAHO cells than chemotherapy. (**D**,**E**) Cells overexpressing SDHA are significantly more sensitive to shikonin than respective controls. In cell lines overexpressing SDHA shikonin reduced cell viability more profoundly than carboplatin in both cell lines, and than paclitaxel in Caov3 cell line. (**A**–**E**) Data are represented as mean ± SEM; * = *p* < 0.05, *** = *p* < 0.001, **** = *p* < 0.0001, and NS = not significant (one-way ANOVA followed by post hoc Tukey’s test). For additional information see Supplementary Table S2 and Figure S4.

We reasoned that SDHA overexpressing cells could be particularly vulnerable to drugs disrupting glucose and/or glutamine metabolism. To identify agents specifically targeting the SDHA overexpressing ovarian cancer cells, we utilized a custom compound library (from Seleckchem) composed of 64 anti-metabolic compounds (Supplementary Table S2). First, we screened the anti-metabolic agents across 4 ovarian cancer cell lines, including those endogenously overexpressing SDHA, and those characterized by naturally low SDHA expression levels. The cell lines were exposed to 1 μM dose of each compound for 4 days followed by the assessment of cell viability. The results showed that 15 compounds hadno effect on cell viability (100% viable cells), 42 compounds moderately reduced cell viability (99–50% viable cells), and the remaining 7 compounds strongly reduced cell viability (49–15% viable cells) as shown on Figure 8A.

Next, we selected 7 the most potent anti-metabolic compounds and evaluated their efficacy in SDHA-high vs. SDHA-low cell lines. We observed that both shikonin and methotrexate significantly reduced viability of SDHA-high cell lines when compared with SDHA-low counterparts, however the shikonin was the most effective agent in specifically killing SDHA overexpressing cells (Figure 8B). Further studies revealed that in vitro treatment of adherent cell cultures with 1 μM of shikonin induces apoptosis more potently in SDHA overexpressing cells than controls as assessed by WES (ProteinSimple) analysis of an apoptotic marker cleaved PARP (Supplementary Figure S5A). Shikonin (extracted from *Lithospermum erythrorhizon* plant) is a Chinese herbal medicine that has been known for its anti-inflammatory and anti-microbial activity [40]. More recently, it has been shown that shikonin exhibits various potent anti-cancer activities including perturbation of purine metabolism, disruption of amino acid biosynthesis and metabolism (e.g., reduction of intracellular glutamine levels), and inhibition of glycolysis [19,20]. Next, we investigated the anti-tumor efficacy of shikonin in vitro and compared it with the efficacy of chemotherapy drugs (carboplatin and paclitaxel) that are commonly used as standard of care in ovarian cancer. The results revealed that shikonin is significantly more effective in killing OVSAHO cells (endogenously overexpressing SDHA) than chemotherapy drugs (Figure 8C). The ovarian cancer cell lines conditionally overexpressing SDHA (Caov3-SDHA and OVCAR3-SDHA) were also significantly more sensitive to shikonin than respective controls. Moreover, in cell lines overexpressing SDHA, shikonin reduced cell viability more profoundly than carboplatin. Paclitaxel and shikonin showed a similar anti-tumor activity against (OVCAR3 cell lines +/− SDHA), however shikonin was significantly more potent in killing Caov3-SDHA cells than paclitaxel (Figure 8D,E and Supplementary Figure S5B–D).

Collectively, we identified a highly potent compound shikonin that effectively kills SDHA overexpressing ovarian cancer cells. The SDHA overexpression strongly sensitized cancer cells to shikonin, which exhibited a profound anti-tumor efficacy superior that seen with traditional chemotherapy.

## 4. Discussion

The reprograming of cellular metabolism is an essential mechanism of tumorigenesis. The metabolic plasticity allows cancer cells to adapt to various unfavorable microenvironmental conditions and sustain uncontrolled cell proliferation, which promotes tumor progression and metastasis [1,41]. In this study, we identified a mitochondrial enzyme succinate dehydrogenase SDHA as a key protein associated with an elevated mitochondrial metabolism in ovarian tumors. The SDHA gene amplification is highly prevalent in ovarian cancer, and has been reported in a considerably higher frequency in ovarian tumors than in many other malignancies (TCGA data) indicating its potential role in reprogramming of ovarian cancer metabolism.

Succinate dehydrogenase has been previously studied in the context of its deficiency in some rare disorders and malignancies [11,14,15,16,17]. However, the SDHA gain-of-function studies are sparse. Increased SDHA function has been associated with elevated mitochondrial respiration and fumarate accumulation driving pathological metabolism in certain diseases [42,43,44]. For instance, in patients with primary antibody deficiency syndrome, the SDHA gain-of-function phenotype leads to an inflammatory reprogramming of lymphocytes B promoting systemic inflammation and worsening the severity of the disease [42]. In metastatic uveal melanoma, elevated SDHA contributes to a metabolic dysregulation with increased mitochondrial respiration, which leads to resistance to therapy, and a significantly shorter time to metastasis and death of patients [44].

In the present study, our initial findings highlighted a potential importance of SDHA upregulation in ovarian cancer and set the stage for further more rigorous research. First, we performed an analysis of SDHA expression in a collection of healthy fallopian tubes and HGSOC PDX models, which showed that SDHA protein levels are significantly lower in fallopian tubes than in PDX tumors. Further, we observed that ovarian PDXs and established ovarian cancer cell lines exhibit a range of SDHA expressions. Importantly, the percentage (23.5%) of SDHA-high PDX tumors in our data set is consistent with the percentage (19%) of ovarian tumors harboring amplification and/or overexpression of SDHA in patients population (TGCA data set) [24]. Our findings are also in agreement with the study by Guo et al., who reported that SDHA is commonly upregulated in ovarian cancer cell lines [43]. As a next step, we functionally validated our ovarian cancer models and showed that the conditional overexpression of SDHA in ovarian cancer cell lines significantly increased succinate dehydrogenase enzyme activity, while SDHA knockdown suppressed its activity. In addition, the increased succinate dehydrogenase activity in those cells was associated with fumarate accumulation. Fumarate accumulation in cells with upregulated SDHA has been also reported in other diseases. For instance, Burgener et al., showed an increased fumarate levels in lymphocytes B with SDHA gain-of-function phenotype in patients with primary antibody deficiency syndrome [42].

To evaluate the SDHA biological functions in ovarian cancer, we assessed the effect of SDHA overexpression on cell proliferation and in vivo tumor growth. The in vitro experiments revealed that SDHA upregulation is associated with a reduced cell proliferation in some cell lines, while in other cell lines the proliferation of cells is only marginally affected. In vivo data showed a tendency towards reduced tumor growth rate in mice bearing OVCAR3 tumor model. Our findings are in agreement with published studies. For instance, Xu et al., reported that the overexpression of SDHA in renal carcinoma cells inhibited cell proliferation in vitro and suppressed tumor growth in a nude mouse model in vivo [45]. Different study showed that a high expression of SDHA inhibited cell proliferation and invasion of multiple myeloma cell lines in vitro [46].

Further, our work demonstrated that SDHA overexpression is associated with a generation of significantly more and larger cancer cell colonies in anchorage-independent conditions. It has been shown that aggressive and metastatic tumor cells are able to survive and rapidly propagate in the absence of anchorage to the extracellular matrix, while less tumorigenic or normal cells undergo growth inhibition and/or apoptosis (a process known as anoikis) [47]. These data indicate that while SDHA overexpressing cells proliferate slower, these cells have improved ability to survive and generate colonies in suspension within a semi-solid matrix. This is highly relevant to ovarian cancer biology, since this tumor type develops large amounts of ascitic fluid in the peritoneal cavity containing floating cancer cells that have to acquire the ability to survive, propagate and metastasize in order to promote disease progression. Other studies reported that the upregulation of SDHA inhibited migration and invasion of renal carcinoma cells [45]. Similarly, Sun et al., reported that SDHA overexpressing multiple myeloma cells are less invasive than control cells [46]. Based on this collective knowledge, we propose that SDHA overexpression could promote cell survival and ability to proliferate in anchorage-independent conditions, which is in contrast to SDHA-low tumor cells that more efficiently proliferate, migrate and invade in adherent cell cultures.

In the present study, we set out to investigate the differences in bioenergetic profiles of ovarian cancer cell lines overexpressing SDHA vs. their respective SDHA-low counterparts. The cell lines showed a substantial diversity in their bioenergetic requirements, which was associated with the SDHA overexpression status. Our data consistently demonstrated that the cell lines with SDHA overexpression showed a significantly higher mitochondrial respiration, and tended to have higher maximal and reserve glycolytic capacity. This highly metabolically active phenotype of SDHA overexpressing cells was reflected by significantly increased total ATP yield. Variability in bioenergetic profiles and metabolic plasticity have been previously described in ovarian cancer and other tumor types by others [8,21,32,48,49]. For instance, Dar et al., reported that metabolic changes in ovarian cancer occur as a result of chemotherapy, where the cells acquire metabolic flexibility to survive a chemotherapy insult [32]. Other studies identified a subgroup of highly metabolically active ovarian cancer cells characterized by enhanced OXPHOS, high glucose uptake, and high glycolysis. The high bioenergetic signature of these cells improved cell ability to form spheroids and survive in anchorage-independent conditions [8,21], similarly to our current findings. In addition, high energy phenotype seems to be a feature of ovarian tumor cells, since normal ovarian epithelial cells exhibit low mitochondrial respiration and glycolytic rates when compared with several distinct ovarian cancer cell lines [21].

In our study, we also noted that all cell lines regardless of SDHA status preferentially used glycolysis pathway to fulfill their energy requirements as assessed by higher rates of ATP production from glycolysis than from OXPHOS, which is also in agreement with published data [50,51,52]. Sun et al., reported that the growth of ovarian cancer cells relies primarily on glucose and glycolysis, which is the most important pathway for energy generation and cell survival [52]. In a different study, Creekmore et al., observed that the metabolic phenotype of ovarian cancer cells changed to a more glycolytic as the cell phenotype progressed from a benign to a highly aggressive [50]. Similarly, in a murine ovarian cancer model, the late stage tumor cells showed an increased utilization of glycolysis pathway [51]. In agreement with the above, our findings support the concept that ovarian cancer cells with high glycolysis are also able to efficiently utilize OXPHOS following SDHA overexpression, which is manifested by enhanced energy metabolism.

In different sets of experiments, we deprived ovarian cancer cells of glucose or glutamine to determine if SDHA overexpression redirects cellular metabolism in response to nutrient deficiency. The cells showed substantial metabolic plasticity switching between glycolysis and oxidative phosphorylation to adapt to changing nutrient conditions. We observed that glucose limitation significantly reduced ATP production from glycolysis that has been compensated with a higher ATP yield from OXPHOS, while glutamine deprivation increased glycolytic ATP yield suppressing ATP production via mitochondrial respiration. Such metabolic flexibility allows cells to become more resilient in variable conditions of tumor microenvironment [8,9,32,53]. In a study by Anderson et al., the authors reported a highly adaptable metabolic phenotype of mouse ovarian tumor cells that were enriched in the stem cell population. The cells were able to increase OXPHOS and glycolysis under appropriate stress, whereas parental tumor cells lacking stem cell features exhibited the glycolytic Warburg effect and were not able to efficiently modulate cellular metabolism under stress [53]. In our study, however, all ovarian cancer cell lines showed metabolic plasticity to some degree when exposed to metabolic stress (limitation of glucose or glutamine). We propose that well-established human ovarian cancer cell lines have already acquired sufficient metabolic plasticity to adapt to limited nutrients availability, similarly as mouse ovarian tumor cells with stem cell features. However, our data showed that the ability to compensate for the loss of ATP production following deprivation of respective nutrients is different between cell lines with and without SDHA overexpression. Our findings indicate that SDHA overexpressing cell lines are not able to fully compensate for the loss of ATP production following glucose or glutamine deprivation, which results in a significant drop in a total ATP yield. In contrast, the corresponding control cells demonstrated an improved metabolic capacity allowing them to better maintain a constant ATP production rate in nutrient deprived conditions. These data suggest that SDHA overexpressing cells characterized by elevated energy metabolism can be more sensitive to a deficiency in essential nutrients or cellular stress. Indeed, it has been shown that ovarian cancer cells with high glycolytic and mitochondrial activity displayed strong sensitivity to inhibition of either glycolysis or OXPHOS that led to a bioenergetic dysfunction and cell death [21]. Different study revealed that high-invasive ovarian cancer cell lines with enhanced metabolism were dependent on glutamine, which deprivation was more detrimental to those cells than to the low-invasive counterparts [7]. However, others reported that a highly adaptable metabolic phenotype of cancer cells allow them to survive cellular stress or substrate deficiency [8,32]. Nevertheless, our data and previous studies indicate that certain cancer cells with highly metabolic phenotype and increased energy demands may have diminished ability to cope with nutrients deficiency. Thus, we reasoned that SDHA overexpressing cells could be particularly vulnerable to drugs disrupting glucose and/or glutamine metabolism. We carried out a screen of anti-metabolic agents, and identified a potent drug shikonin that more effectively kills SDHA overexpressing ovarian cancer cells than SDHA-low counterparts. Moreover, the SDHA overexpression strongly sensitized cancer cells to shikonin, which exhibited a profound anti-tumor efficacy superior to that seen with traditional chemotherapy. Shikonin is a traditional Chinese medicine with various biological activities, among which inhibition of glycolysis and glutamine metabolism was reported [19,20]. We propose that SDHA overexpressing cells carry out high energy metabolism and depend on both glucose to perform glycolysis and glutamine to support TCA cycle flux and OXPHOS in mitochondria, thus disruption of both glycolysis and glutamine metabolism could be detrimental for survival of those cells. In fact, it has been previously observed that the combination of glycolysis and glutaminolysis supports rapid proliferation of cancer cells through production of ATP and other biosynthetic precursors, and a simultaneous inhibition of both pathways could be a promising therapeutic strategy [52,54]. For instance, Sun et al., demonstrated that the inhibition of both glutaminolysis and glycolysis (by aminooxyacetate and by 2-DG, respectively) led to a strong synergistic cytotoxic effect on ovarian cancer cell viability [52]. A similar therapeutic approach has been also explored by others, where a combination of metformin (OXPHOS inhibitor) and 2-DG inhibited mitochondrial respiration and glycolysis in prostate [55] and ovarian [56] cancer cells leading to a severe depletion of ATP and cell death.

## 5. Conclusions

In summary, our work provided novel insights into the role of SDHA upregulation in reprogramming of energy metabolism in ovarian cancer. We showed that SDHA overexpressing cells are highly metabolically active, relying on both glycolysis and oxidative phosphorylation to meet their energy needs. While this phenotype may not drive a higher proliferation rate, it improves cells’ ability to survive anchorage-independent conditions. Importantly, we found that ovarian cancer cells with elevated SDHA are more vulnerable to deprivation of both glucose and glutamine, which is associated with a substantial reduction of ATP yield in those cells. Lastly, we implemented a drug screening strategy and identified anti-metabolic compound shikonin, which demonstrated a specific and potent efficacy against SDHA overexpressing ovarian cancer cells. Our future directions will be focused on pre-clinical testing of shikonin as a promising therapeutic approach for SDHA-amplified tumors. This work is a major step towards our long-term goal, to develop innovative ways to precisely target ovarian cancer-specific metabolism to improve treatment options for ovarian cancer patients.

## Data Availability

The data presented in this study are contained within the article or Supplementary Materials. Additional data supporting the findings of this study are available from the corresponding author upon reasonable request.

## Supplementary Materials

The following supporting information can be downloaded at: https://www.mdpi.com/article/10.3390/cancers14205097/s1, Table S1: Analysis of metabolic pathways by RPPA. Table S2: Anti-metabolic compound library screen. Figure S1: Analysis of survival in ovarian cancer patients with w SDHA amplification or overexpression. Figure S2: The effect of SDHA overexpression on cell proliferation and anchorage-independent growth. Figure S3: The impact of SDHA overexpression on ovarian cancer dependence on glucose or glutamine. Figure S4: Anti-metabolic compound library screen in individual cell lines. Figure S5: Drug response assays. Figure S6: Original images for blots.
