# Supplementary material for: Upregulation of Succinate Dehydrogenase (SDHA) Contributes to Enhanced Bioenergetics of Ovarian Cancer Cells and Higher Sensitivity to Anti-Metabolic Agent Shikonin
Source: Cancers (Basel). 2022 Oct 18;14(20):5097. doi: 10.3390/cancers14205097 (PMC9599980; doi:10.3390/cancers14205097)
Supplement: Supplementary file 1 [file cancers-14-05097-s001.zip › Supplementary Figure S1.pdf]

Supplementary Figure S1

cBioPortal FOR CANCER GENOMICS | Modify Query | Ovarian Serous Cystadenocarcinoma (TCGA, Firehose Legacy)
All samples (600 patients / 617 samples) - SDHA

A

Survival Type	Number of Patients	p-Value	q-Value
Overall	568	0.0552	0.110
Disease Free	483	0.524	0.524

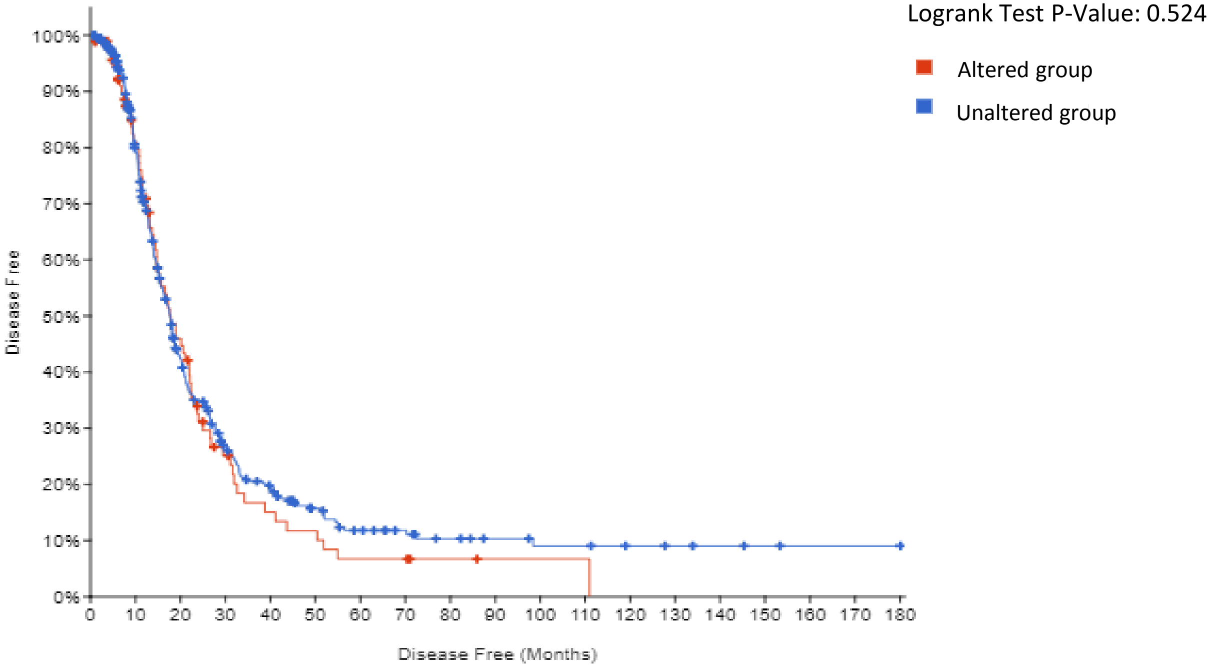

	Number of Cases, Total	Number of Events	Median Months Disease Free (95% CI)
Altered group	93	70	17.64 (14.68 - 22.27)
Unaltered group	390	283	17.71 (16.03 - 18.89)

B

Survival Type	Number of Patients	p-Value	q-Value
Overall	568	0.0552	0.110
Disease Free	483	0.524	0.524

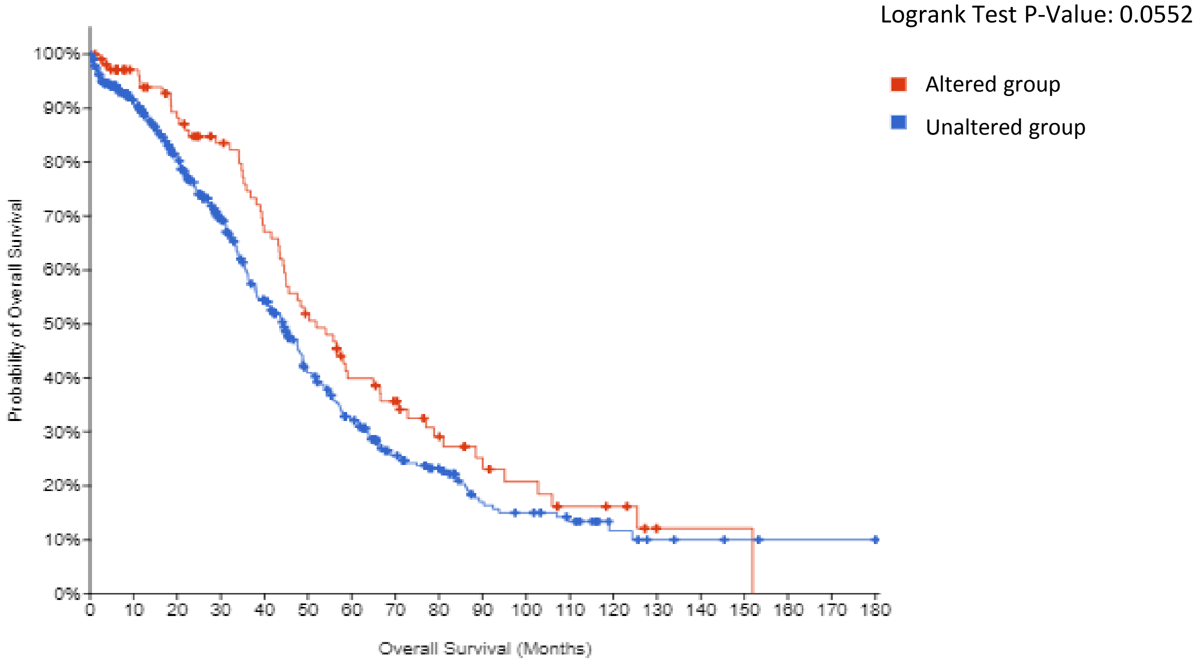

	Number of Cases, Total	Number of Events	Median Months Overall (95% CI)
Altered group	105	64	51.87 (44.51 - 66.52)
Unaltered group	463	277	44.48 (38.40 - 48.72)

Supplementary Figure S1. Analysis of disease free survival (**A**) and overall survival (**B**) by Kaplan-Meier method in ovarian serous cystadenocarcinoma dataset (TCGA). Altered group refers to patients with SDHA amplification or overexpression, while unaltered group represents remaining patients. There is no correlation between SDHA overexpression status and disease free survival. However, there is tendency towards improved overall survival of ovarian cancer patients with SDHA amplification/overexpression.

Supplementary Figure S1

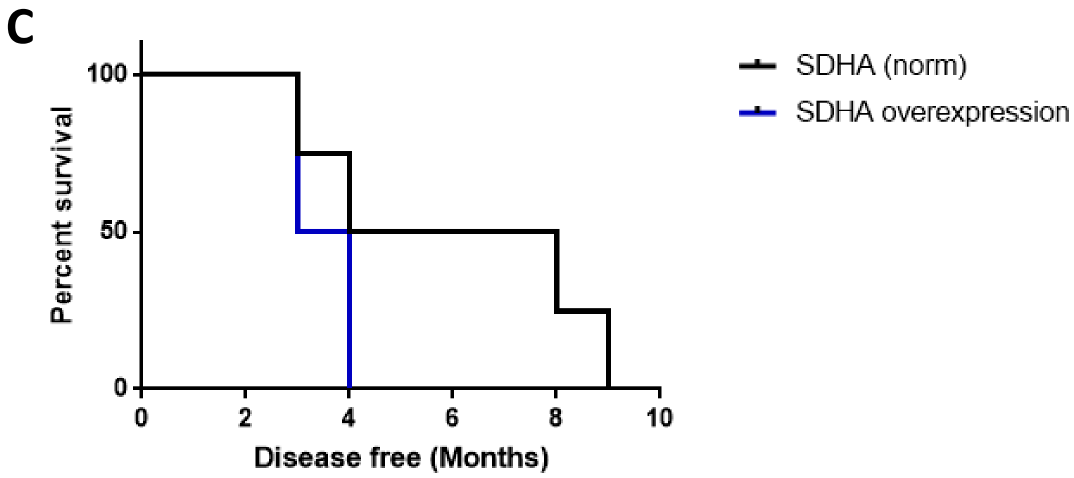

Logrank Test P-Value: 0.2842

Median survival (months)

SDHA (norm)	6	
SDHA overexpression	3.5	
95% CI of ratio	0.314 to 9.36	0.1068 to 3.185

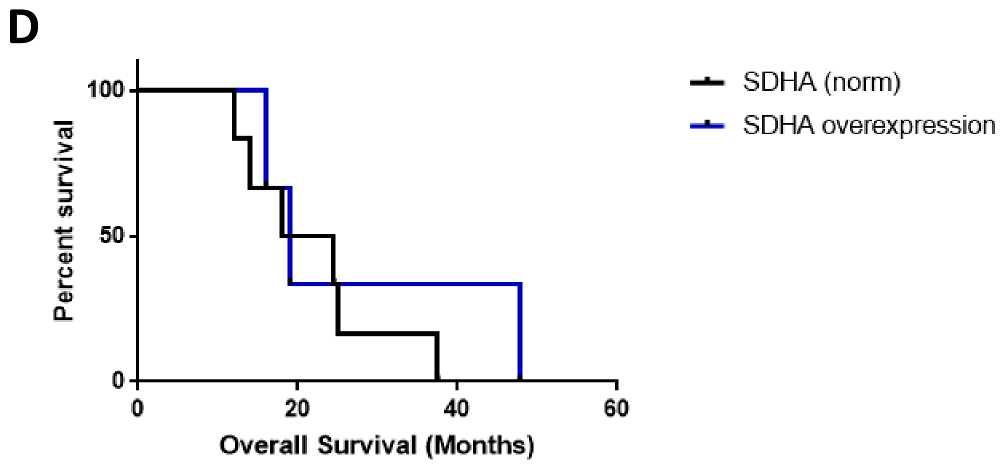

Logrank Test P-Value: 0.4987

Median survival (months)

SDHA (norm)	21.25	
SDHA overexpression	19	
95% CI of ratio	0.2797 to 4.472	0.2236 to 3.575

Supplementary Figure S1. Analysis of disease free survival (**C**) and overall survival (**D**) by Kaplan-Meier method in 9 PDX models representing high-grade serous ovarian carcinoma (HGSOC) segregated into two groups, PDXs with SDHA overexpression, and PDXs with normal (norm) SDHA levels. There is no correlation between SDHA overexpression status and disease free survival or overall survival.
