# Supplementary material for: Upregulation of Succinate Dehydrogenase (SDHA) Contributes to Enhanced Bioenergetics of Ovarian Cancer Cells and Higher Sensitivity to Anti-Metabolic Agent Shikonin
Source: Cancers (Basel). 2022 Oct 18;14(20):5097. doi: 10.3390/cancers14205097 (PMC9599980; doi:10.3390/cancers14205097)
Supplement: Supplementary file 1 [file cancers-14-05097-s001.zip › Supplementary Figure S2.pdf]

Supplementary Figure S2

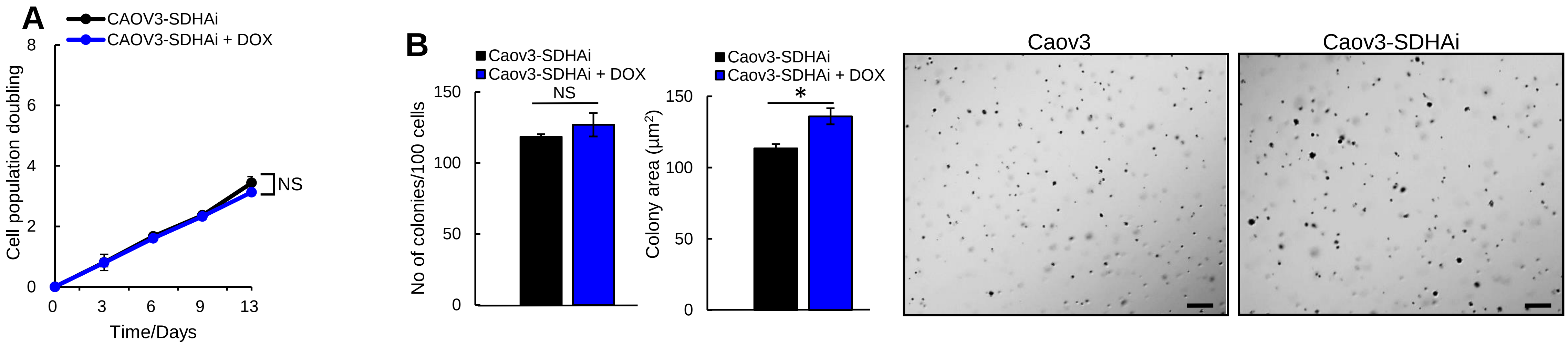

Supplementary Figure S2. A. The effect of inducible SDHA overexpression on the Caov3 cell proliferation was assessed by 3T5 cell doubling assay. **B.** The effect of SDHA overexpression on anchorage-independent growth and cell survival. Images represent anchorage-independent growth and colony formation of Caov3-SDHAi cells stimulated with 100 ng/ml dox. Number and size of cell colonies were quantified and illustrated on respective graphs. Data are expressed as mean ± SEM. Statistical significance of data was assessed unpaired t test. Scale bar is 100 μM.
