# Supplementary material for: Upregulation of Succinate Dehydrogenase (SDHA) Contributes to Enhanced Bioenergetics of Ovarian Cancer Cells and Higher Sensitivity to Anti-Metabolic Agent Shikonin
Source: Cancers (Basel). 2022 Oct 18;14(20):5097. doi: 10.3390/cancers14205097 (PMC9599980; doi:10.3390/cancers14205097)
Supplement: Supplementary file 1 [file cancers-14-05097-s001.zip › Supplementary Figure S3.pdf]

Supplementary Figure S3

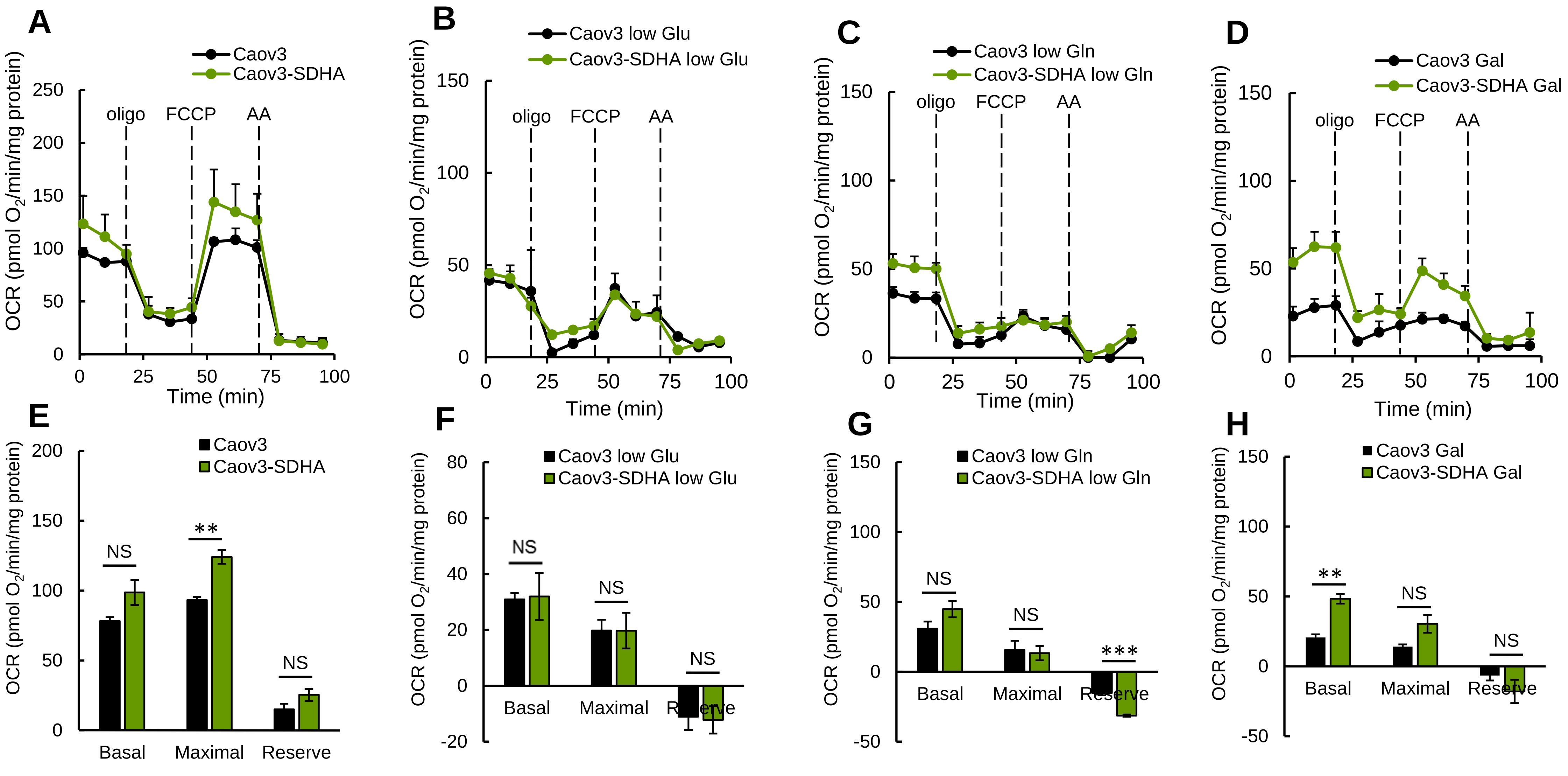

Supplementary Figure S3. The effect of nutrient deprivation on mitochondrial respiration in the Caov3 cell line overexpressing SDHA. (**A-E**) Mitochondrial respiration (OCR) was measured in Caov3 +/-SDHA cell lines in complete medium by Seahorse XF Cell Mito Stress Test. The SDHA overexpression significantly increased maximal respiration and showed tendency to induce basal and reserve respiration. (**B-F**) In low glucose (Glu) medium, the OCR was suppressed in both cell lines, when compared to OCR in complete medium. (**C-G**) In low glutamine (Gln) medium, the OCR was suppressed in both cell lines to similar extend as in low Glu medium. (**D-H**) In galactose (Gal) medium, the OCR was suppressed in both cell lines to similar extend as in low Glu and low Gln medium.

Supplementary Figure S3

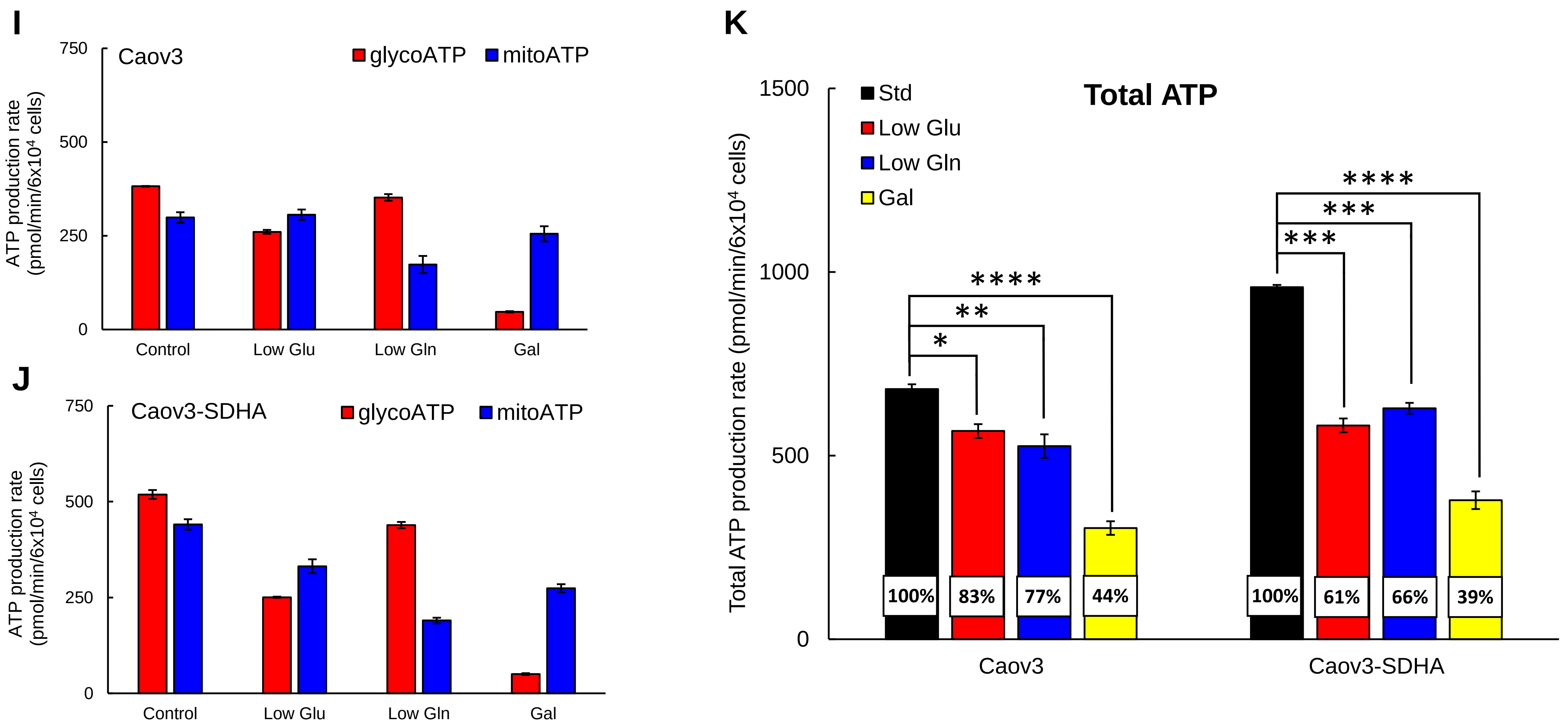

Supplementary Figure S3. The effect of nutrient deprivation on ATP production rate in the Caov3 cell line overexpressing SDHA. (**I-K**) Graph represents quantitative measure of an ATP production rate from mitochondrial OXPHOS (mitoATP) or glycolysis (glycoATP) in various nutrient deprivation conditions in Caov3 cells (**I**) and Caov3-SDHA cells (**J**) as assessed by Seahorse ATP Rate Assay. (**K**) Total ATP production rate in Caov3 cell lines +/- SDHA overexpression deprived from selected nutrients. The total ATP yield was the highest in SDHA amplified cells in standard medium. Limiting amount of glucose or glutamine in culture medium suppressed ATP production rate to 83%, and 77% in Caov3 cells, and to 61%, and to 66% in Caov3-SDHA cells, respectively. Both cell lines showed a substantial suppression of ATP production rate in galactose medium, which inhibits glycolysis.
