# Supplementary material for: Upregulation of Succinate Dehydrogenase (SDHA) Contributes to Enhanced Bioenergetics of Ovarian Cancer Cells and Higher Sensitivity to Anti-Metabolic Agent Shikonin
Source: Cancers (Basel). 2022 Oct 18;14(20):5097. doi: 10.3390/cancers14205097 (PMC9599980; doi:10.3390/cancers14205097)
Supplement: Supplementary file 1 [file cancers-14-05097-s001.zip › Supplementary Figure S4.pdf]

Supplementary Figure S4

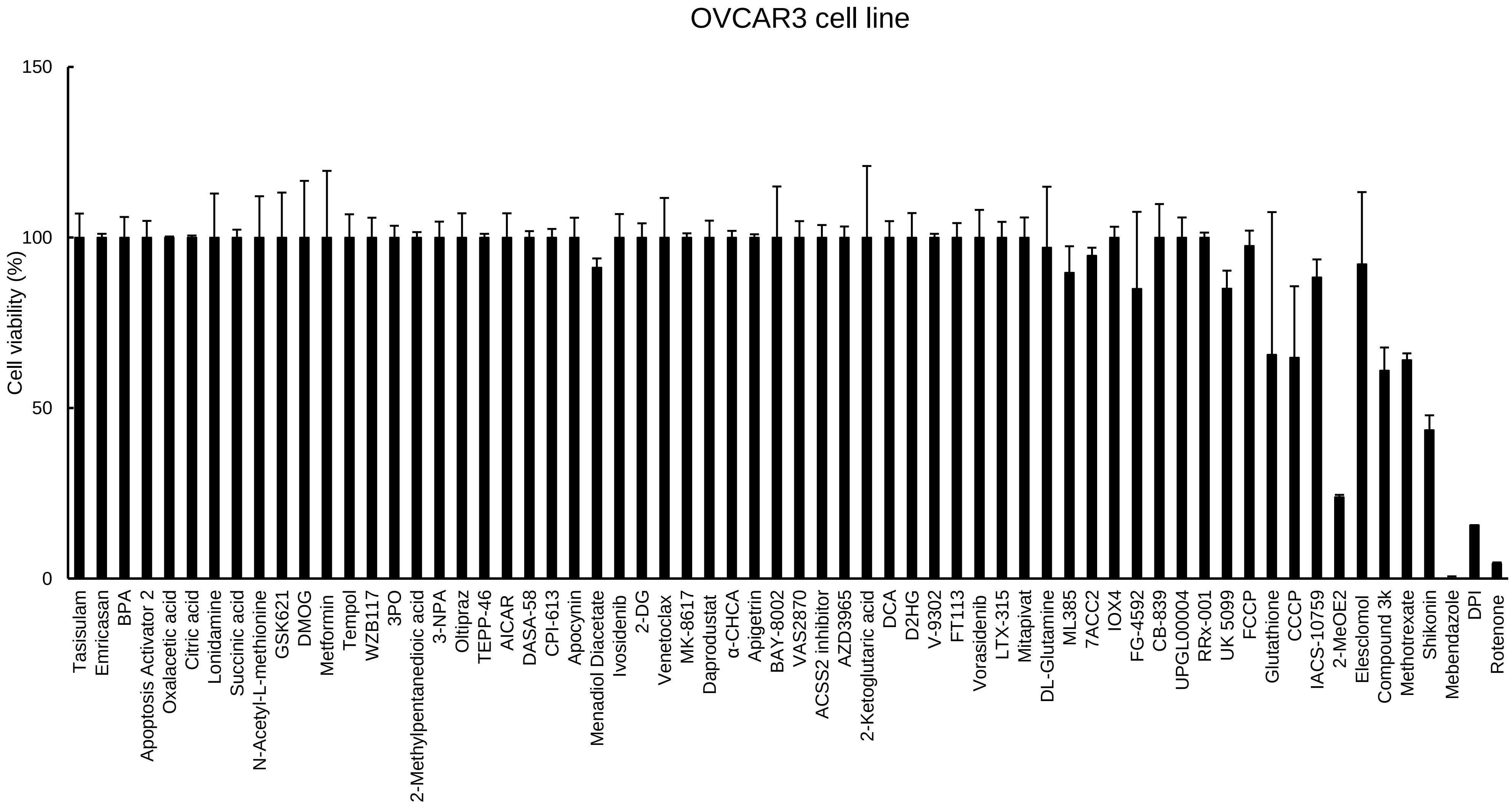

Supplementary Figure S4. Ovarian cancer cell line OVCAR3 was exposed to metabolic compound library. Cells were treated with 1 μM dose of each compound for 4 days followed by MTT assay to assess percentage of viable cells.

Supplementary Figure S4

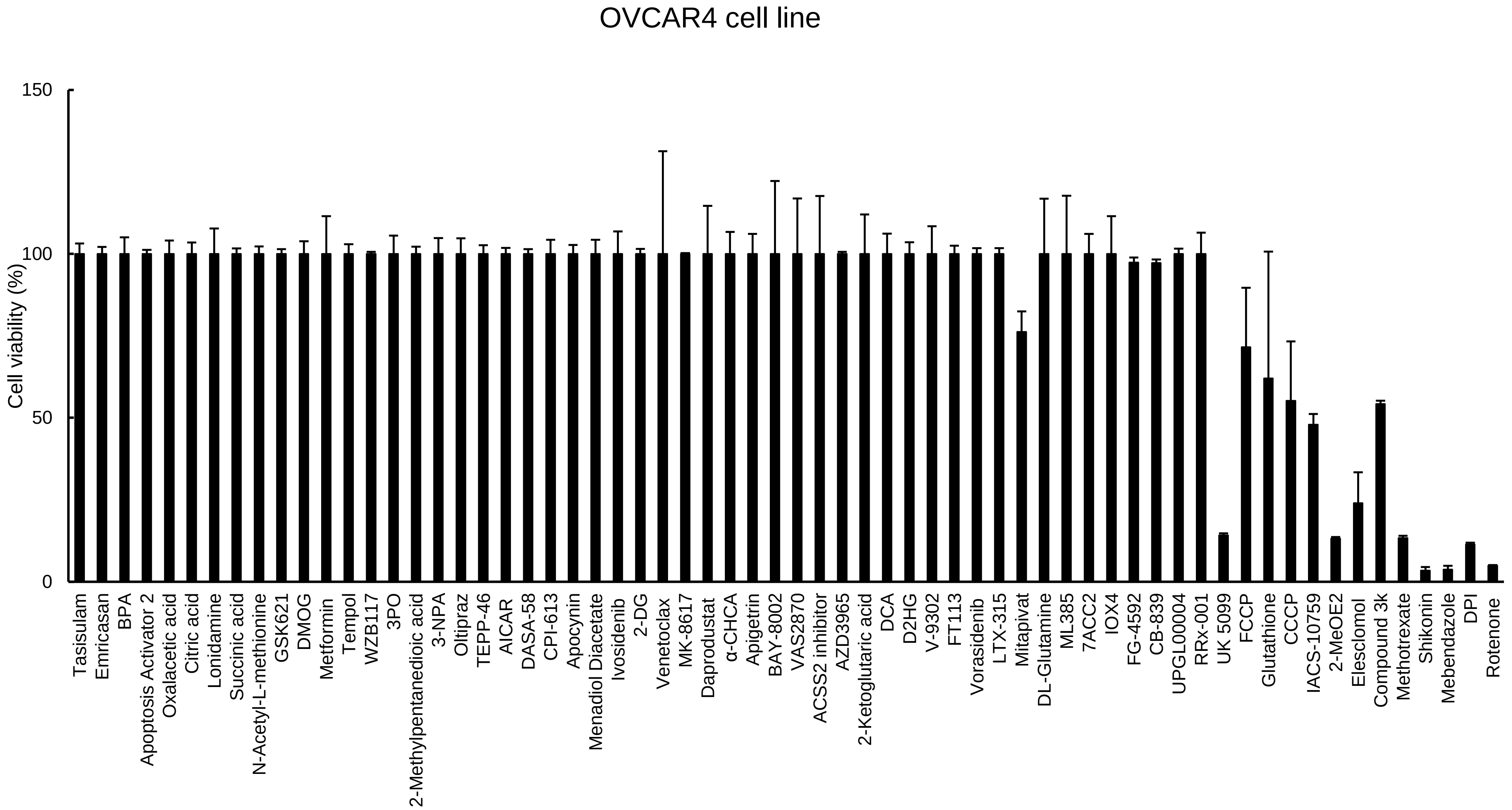

Supplementary Figure S4. Ovarian cancer cell line OVCAR4 was exposed to metabolic compound library. Cells were treated with 1 μM dose of each compound for 4 days followed by MTT assay to assess percentage of viable cells.

Supplementary Figure S4

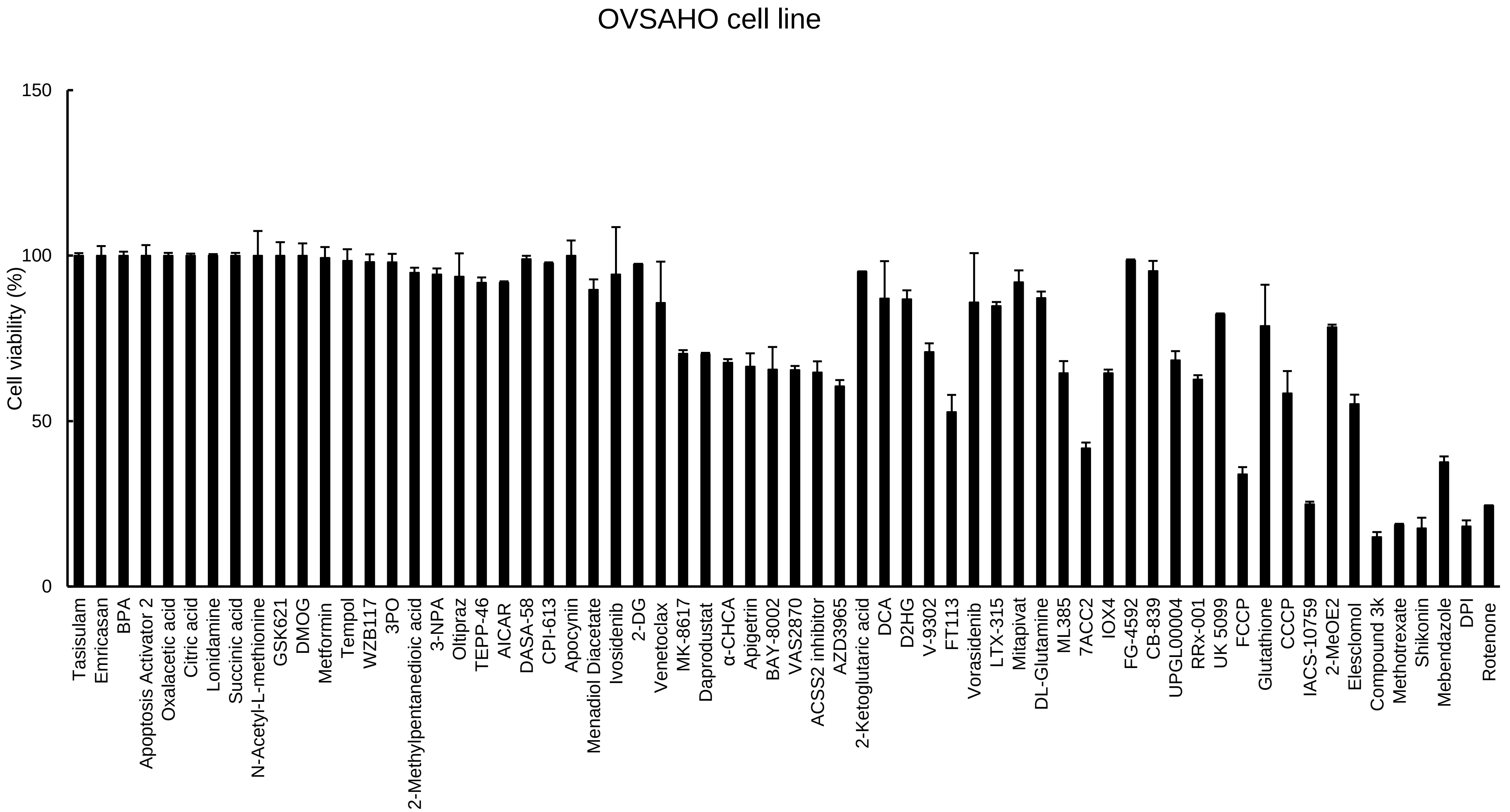

Supplementary Figure S4. Ovarian cancer cell line OVSAHO was exposed to metabolic compound library. Cells were treated with 1 μM dose of each compound for 4 days followed by MTT assay to assess percentage of viable cells.

Supplementary Figure S4

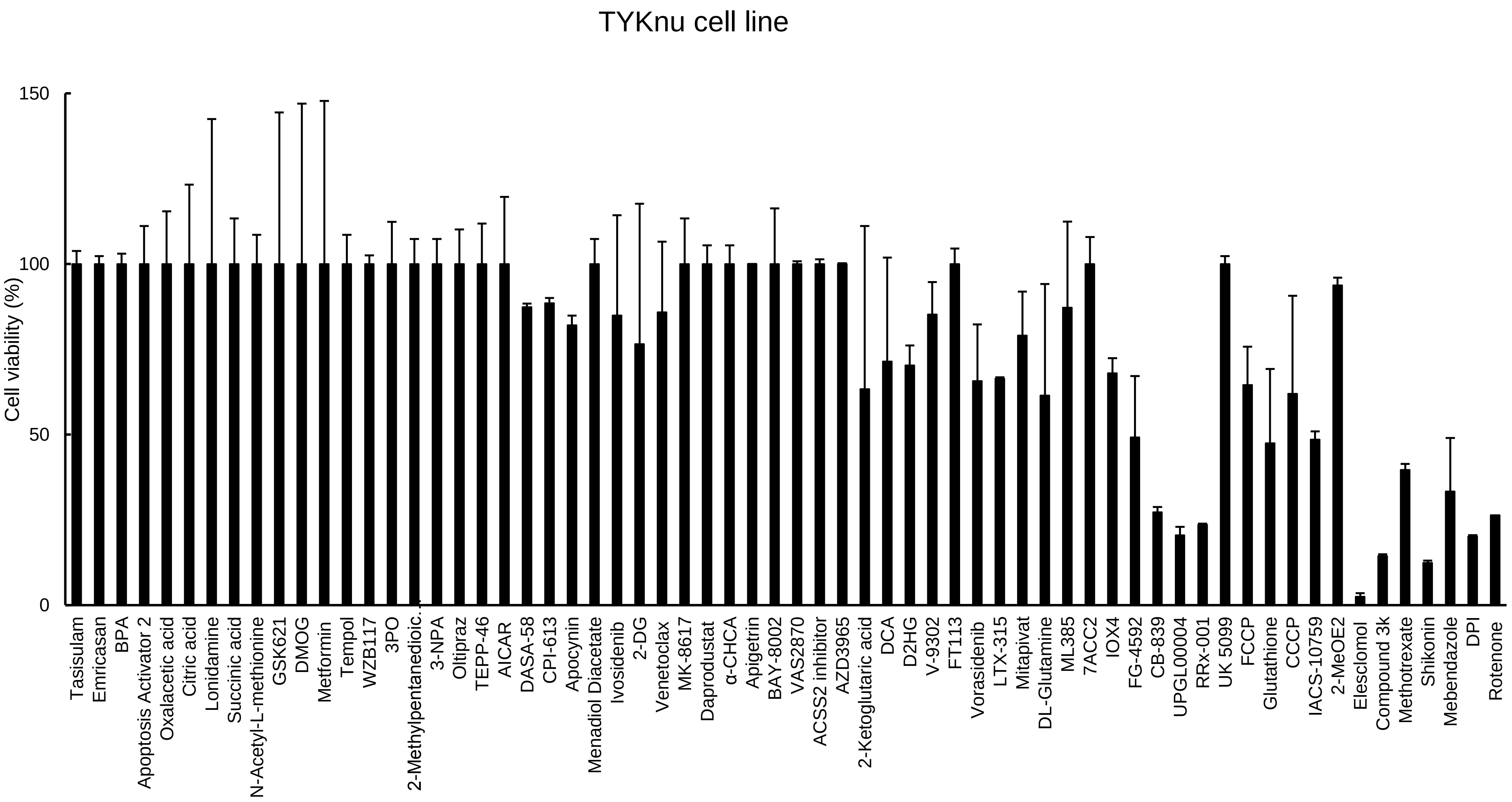

Supplementary Figure S4. Ovarian cancer cell line TYKnu was exposed to metabolic compound library. Cells were treated with 1 μM dose of each compound for 4 days followed by MTT assay to assess percentage of viable cells.
