# Supplementary material for: Upregulation of Succinate Dehydrogenase (SDHA) Contributes to Enhanced Bioenergetics of Ovarian Cancer Cells and Higher Sensitivity to Anti-Metabolic Agent Shikonin
Source: Cancers (Basel). 2022 Oct 18;14(20):5097. doi: 10.3390/cancers14205097 (PMC9599980; doi:10.3390/cancers14205097)
Supplement: Supplementary file 1 [file cancers-14-05097-s001.zip › Supplementary Figure S5.pdf]

Supplementary Figure S5

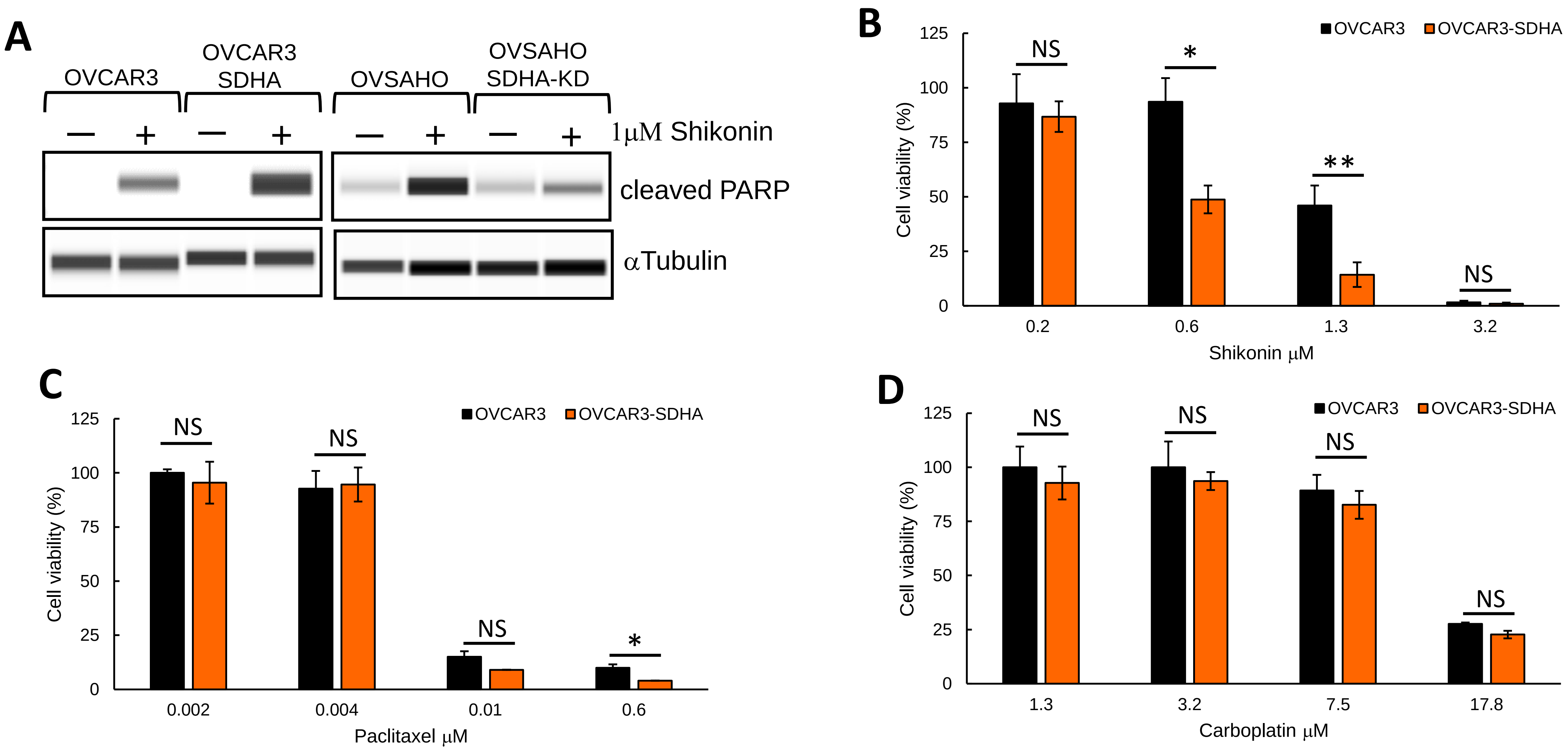

Supplementary Figure S5. Therapeutic response of SDHA overexpressing cell lines vs. controls to shikonin, paclitaxel, and carboplatin. A. WES analysis revealed that *in vitro* treatment with 1 μM of shikonin of adherent cell cultures induces apoptosis more potently in SDHA overexpressing cells than controls (apoptotic marker, cleaved PARP). **B-D.** OVCAR3 cell lines with and without SDHA overexpression were exposed to various doses of shikonin (**B**), paclitaxel (**C**), and carboplatin (**D**). Cell viability was assessed by MTT assay. Data are represented as mean ± SD, unpaired t test.
