# Supplementary material for: Upregulation of Succinate Dehydrogenase (SDHA) Contributes to Enhanced Bioenergetics of Ovarian Cancer Cells and Higher Sensitivity to Anti-Metabolic Agent Shikonin
Source: Cancers (Basel). 2022 Oct 18;14(20):5097. doi: 10.3390/cancers14205097 (PMC9599980; doi:10.3390/cancers14205097)

Figure 2 – WES blots

Figure 2A

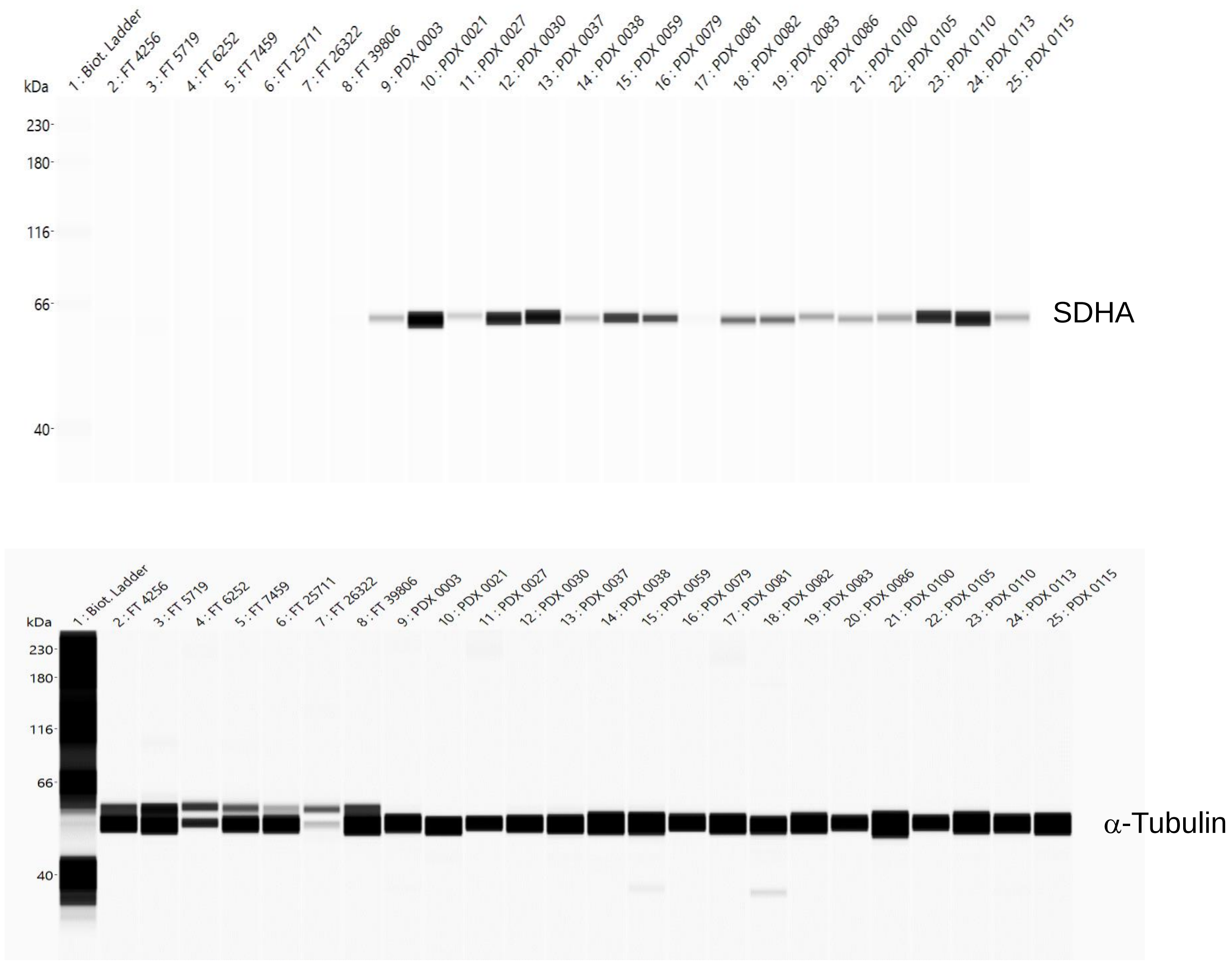

Figure 2B

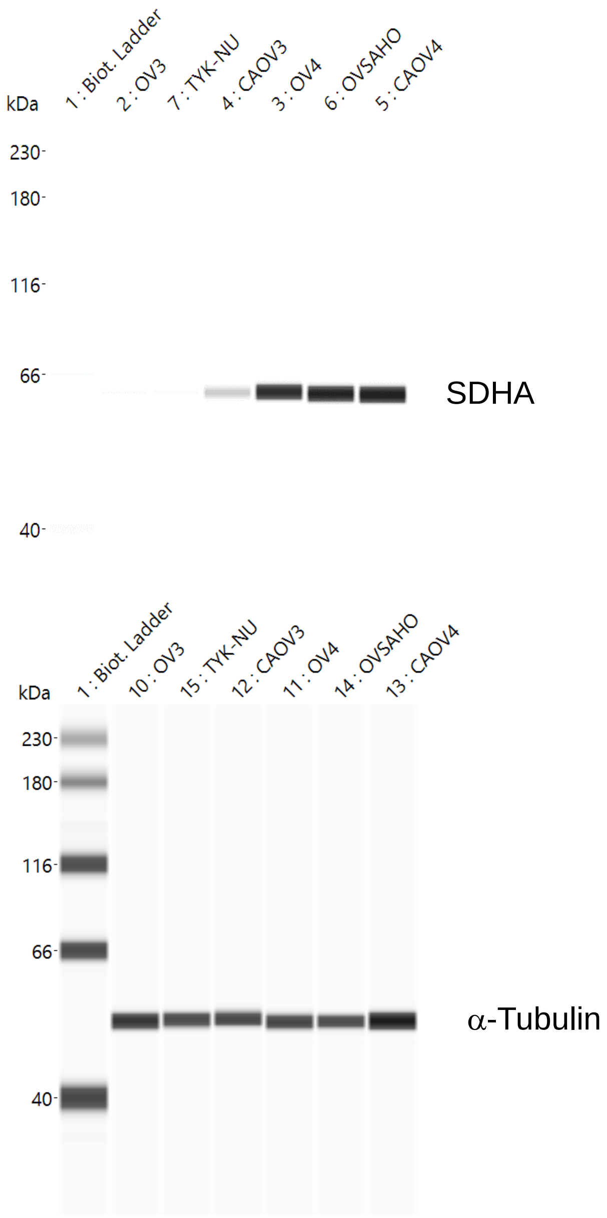

Figure 2 – WES blots

Figure 2C

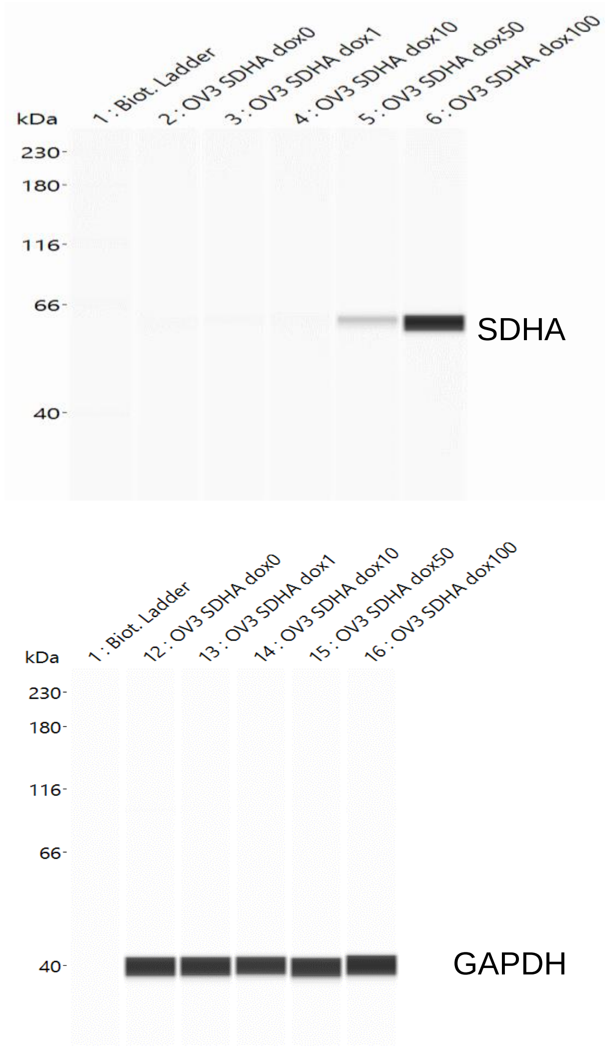

Figure 2D

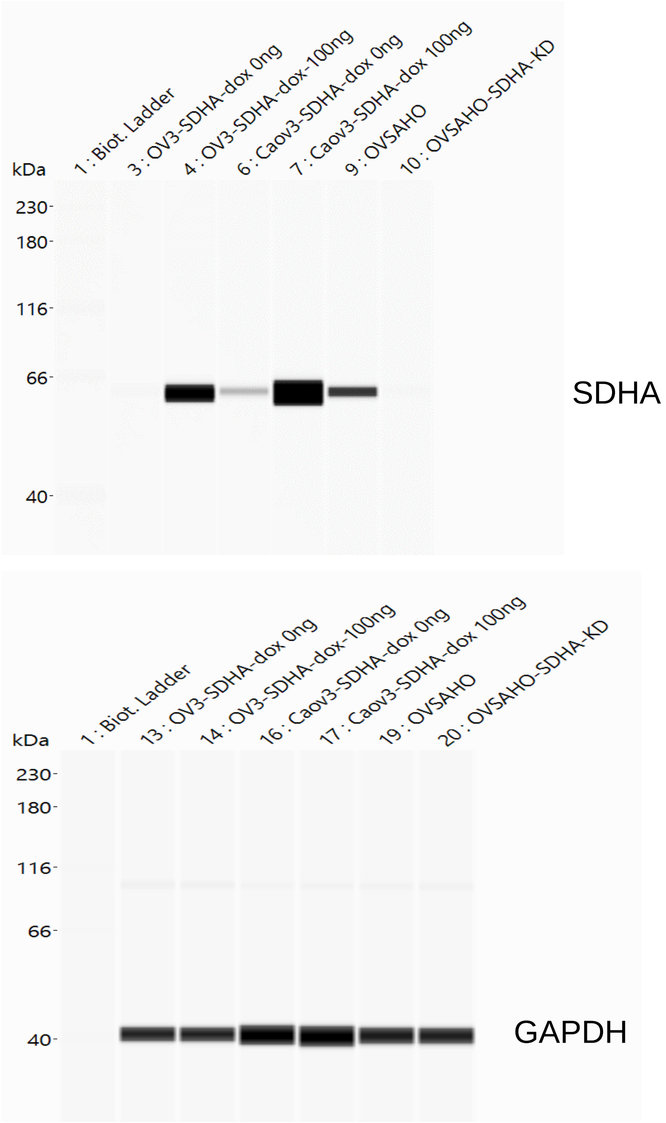

Figure 3 – WES blots

Figure 3D

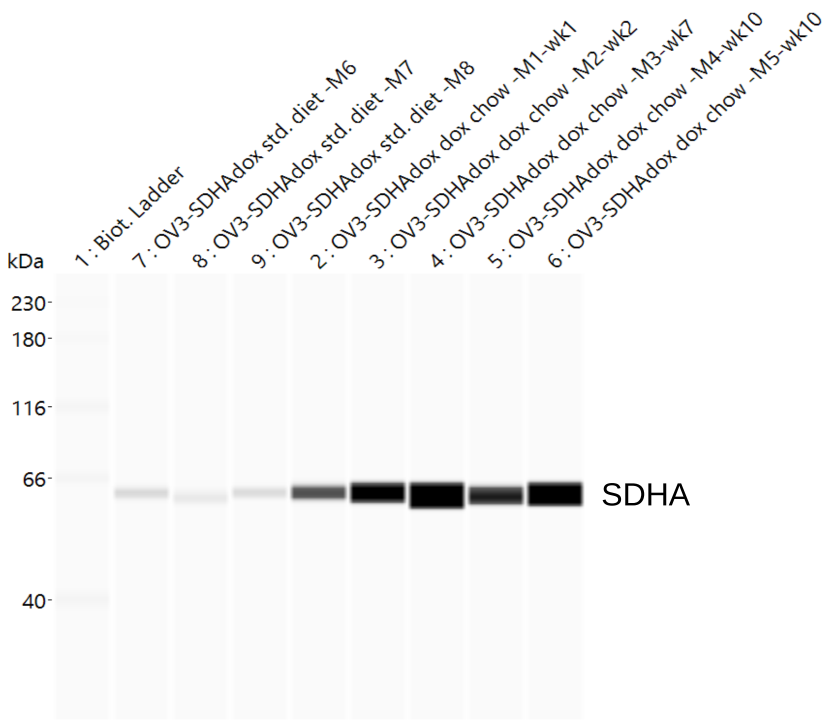

Figure 3D

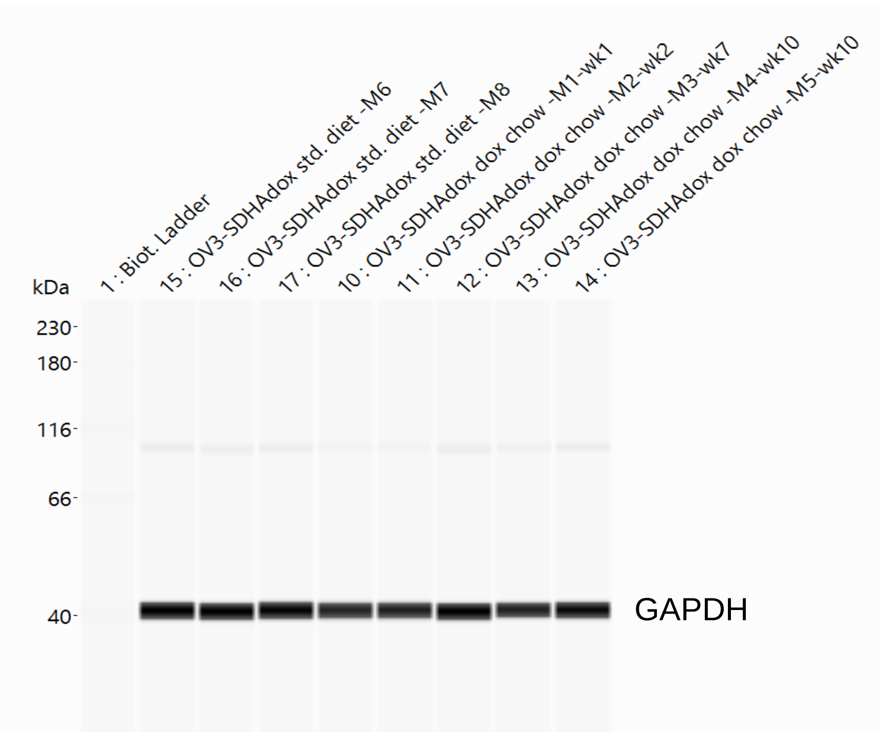

Supplement: Supplementary file 1 [file cancers-14-05097-s001.zip › Supplementary Figure S6.pdf]
